# N^6^-methyladenosine–mediated overexpression of long noncoding RNA *ADAMTS9-AS2* triggers neuroblastoma differentiation via regulating LIN28B/*let-7*/MYCN signaling

**DOI:** 10.1172/jci.insight.165703

**Published:** 2023-11-22

**Authors:** Yun Liu, Jun Zhang, Fang Cao, Xiaobao Dong, Jie Li, Yanna Cao, Zhanglin Li, Yan Guo, Jie Yan, Yuanyuan Liu, Qiang Zhao

**Affiliations:** 1 Department of Pediatric Oncology, Tianjin Medical University Cancer Institute and Hospital, National Clinical Research Center for Cancer, Tianjin’s Clinical Research Center for Cancer, Key Laboratory of Cancer Prevention and Therapy, and; 2 Department of Genetics, School of Basic Medical Sciences, Tianjin Medical University, Tianjin, China.; 3 Department of Thoracic Surgery, The Second Hospital of Tianjin Medical University, Tianjin, China.

**Keywords:** Oncology, Noncoding RNAs

## Abstract

Neuroblastomas have shed light on the differentiation disorder that is associated with spontaneous regression or differentiation in the same tumor at the same time. Long noncoding RNAs (lncRNAs) actively participate in a broad spectrum of biological processes. However, the detailed molecular mechanisms underlying lncRNA regulation of differentiation in neuroblastomas remain largely unknown. Here, we sequenced clinical samples of ganglioneuroma, ganglioneuroblastoma, and neuroblastoma. We compared transcription profiles of neuroblastoma cells, ganglion cells, and intermediate state cells; verified the profiles in a retinoic acid–induced cell differentiation model and clinical samples; and screened out the lncRNA ADAMTS9 antisense RNA 2 (*ADAMTS9-AS2*), which contributed to neuroblastoma differentiation. *ADAMTS9-AS2* upregulation in neuroblastoma cell lines inhibited proliferation and metastatic potential. Additional mechanistic studies illustrated that the interactions between *ADAMTS9-AS2* and LIN28B inhibited the association between LIN28B and primary let-7 (*pri-let-7*) miRNA, then released *pri-let-7* into cytoplasm to form mature *let-7*, resulting in the inhibition of oncogene MYCN activity that subsequently affected cancer stemness and differentiation. Furthermore, we showed that the observed differential expression of *ADAMTS9-AS2* in neuroblastoma cells was due to N^6^-methyladenosine methylation. Finally, *ADAMTS9-AS2* upregulation inhibited proliferation and cancer stem-like capabilities in vivo. Taken together, these results show that *ADAMTS9-AS2* loss leads to malignant neuroblastoma by increasing metastasis and causing dysfunctional differentiation.

## Graphical abstract

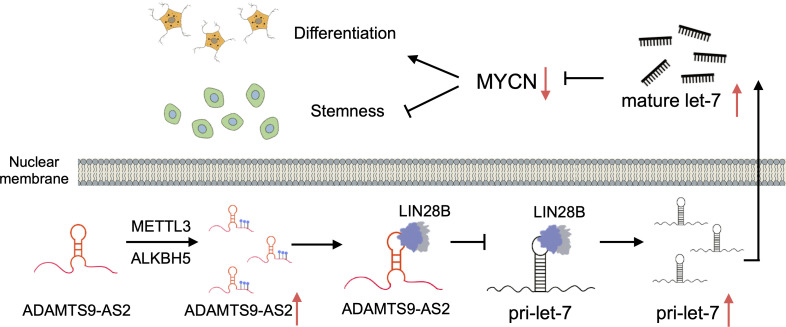

## Introduction

Neuroblastoma is an embryonic malignant tumor caused by improper differentiation of neuronal precursor cells and is the most common malignant extracranial solid tumor in infancy and early childhood (1). The characteristics of children with high-risk neuroblastoma include age greater than 18 months, distal metastasis at the clinical stage, undifferentiated pathological stage, MYCN amplification, and loss of heterozygosity in chromosome 11q23 (2). Over the past decades, analysis of clinical neuroblastoma patients revealed that the neuroblastic differentiation stage influences the prognosis of neuroblastoma. Additionally, clinical manifestations in children with neuroblastoma are diverse due to differences in the degree of differentiation of neuroblastoma cells, which are responsible for the unique heterogeneity observed in neuroblastoma. Although a few neuroblastomas have the potential to spontaneously differentiate and regress, a considerable number of patients are diagnosed with a high-risk form of the disease, where highly intensive combination therapy fails to control progression and recurrence. Meanwhile, little is known about the mechanisms underlying this process. Therefore, exploring the mechanisms of abnormal cell differentiation during neuroblastoma development and identifying key molecular targets for inducing cell differentiation will help in the development of adjuvant therapies for current treatment regimens.

Neuroblastomas are a group of tumors of the sympathetic nervous system and adrenal medulla that originate from primordial neural crest cells. According to the International Neuroblastoma Pathology Classification, these tumors can be divided into neuroblastoma, ganglioneuroblastoma-intermixed, ganglioneuroblastoma-nodular, and ganglioneuroma maturing subtypes (3, 4). The tumor cell component of neuroblastoma is immature neuroblasts, the cells in ganglioneuroma are differentiated and mature ganglion cells, and ganglioneuroblastoma is composed of immature neuroblasts and differentiated mature ganglion cells, which is an intermediate form of neuroblastoma and ganglioneuroma based on its morphology, clinical presentation, and prognosis. The extent of neuroblastic differentiation toward ganglion cells is recognized as the most important morphological feature for predicting the prognosis of neuroblastoma. This characteristic is closely associated with the differentiation disorder, and it is mainly caused by the inhibition of neural stem cell differentiation into ganglion cells and Schwann cells, resulting in immature neuroblastoma and ganglion neuroblastoma (3, 4). In clinical practice, tumors from the same neuroblastoma may contain cells with different degrees of differentiation, for example, neuroblasts with stem cell characteristics, mature ganglion cells, and ganglion neuroblasts. Therefore, tumors from the same neuroblastoma can be used as a model for studying the differentiation mechanism.

Pediatric tumors, especially neuroblastoma, showed the paucity of mutations; additional mechanisms should be discovered to explain the tumorigenesis of neuroblastoma. Recently, transcriptomic studies suggested that epigenetic regulation plays a very important role in the development of these tumors, which mainly includes DNA methylation, RNA methylation, histone modifications, noncoding RNAs, and chromosome remodeling. Approximately 75% of the sequences in the human genome can be transcribed into RNA. Only 1%–2% of the RNAs encode proteins, with the rest existing in the form of noncoding RNA (5). This points to the importance of noncoding RNAs in biological evolution (6, 7). Researchers have shown that long noncoding RNAs (lncRNAs) are associated with many biological functions, including telomere maintenance, transcriptional interference, posttranscriptional regulation, translation control, epigenetic modification, and so on (8). Recent studies have implicated that dysregulated lncRNAs play important roles in cell differentiation. LncRNAs play important roles in muscle differentiation, cardiovascular lineage commitment (9, 10), osteoblast differentiation (11), myoblast differentiation (12), and mesendodermal differentiation (13), indicating that they may be potentially associated with essential developmental regulatory genes. Moreover, ectopic lncRNA expression has been identified in multiple cancers, and lncRNA-based prognostic markers for predicting patient survival and tumor stratification have been suggested (14, 15). Several lncRNAs, including HULC, MALAT1, PCAT-1, and HOTAIR, have been implicated in tumorigenesis (16–18). However, apart from a few lncRNAs such as NBAT-1 (19) and lncNB1 (20), little is known about the role of lncRNAs in neuroblastoma. Therefore, elucidating lncRNA-dependent regulatory mechanisms may improve our understanding of cancer initiation and progression.

In this study, we performed high-throughput transcriptomic analysis of neuroblastoma, ganglioneuroma, and ganglioneuroblastoma samples and identified the lncRNA ADAMTS9 antisense RNA 2 (*ADAMTS9-AS2*) as a critical regulator in the differentiation of neuroblastoma. *ADAMTS9-AS2* expression level was positively correlated with differentiation and negatively correlated with the clinical stage of neuroblastoma. *ADAMTS9-AS2* bound to LIN28B, attenuating the interaction between primary *let-7* (*pri-let-7*) and LIN28B and leading to the release of mature *let-7* microRNA (miRNA), which inhibits oncogene MYCN activity. MYCN is essential for the inhibition of neuronal differentiation (21, 22) and has emerged as a critical factor in the acquisition and maintenance of stem cell properties (23). Our results reveal that *ADAMTS9-AS2* promotes neuronal differentiation and inhibits cancer stemness by directly binding to LIN28B and modulating MYCN activity. Thus, *ADAMTS9-AS2* is a potential marker of differentiation that can be used for risk assessment and targeted for treatment.

## Results

### ADAMTS9-AS2 is differentially expressed in neuroblastoma and is positively correlated with differentiation.

**Figure 1:**
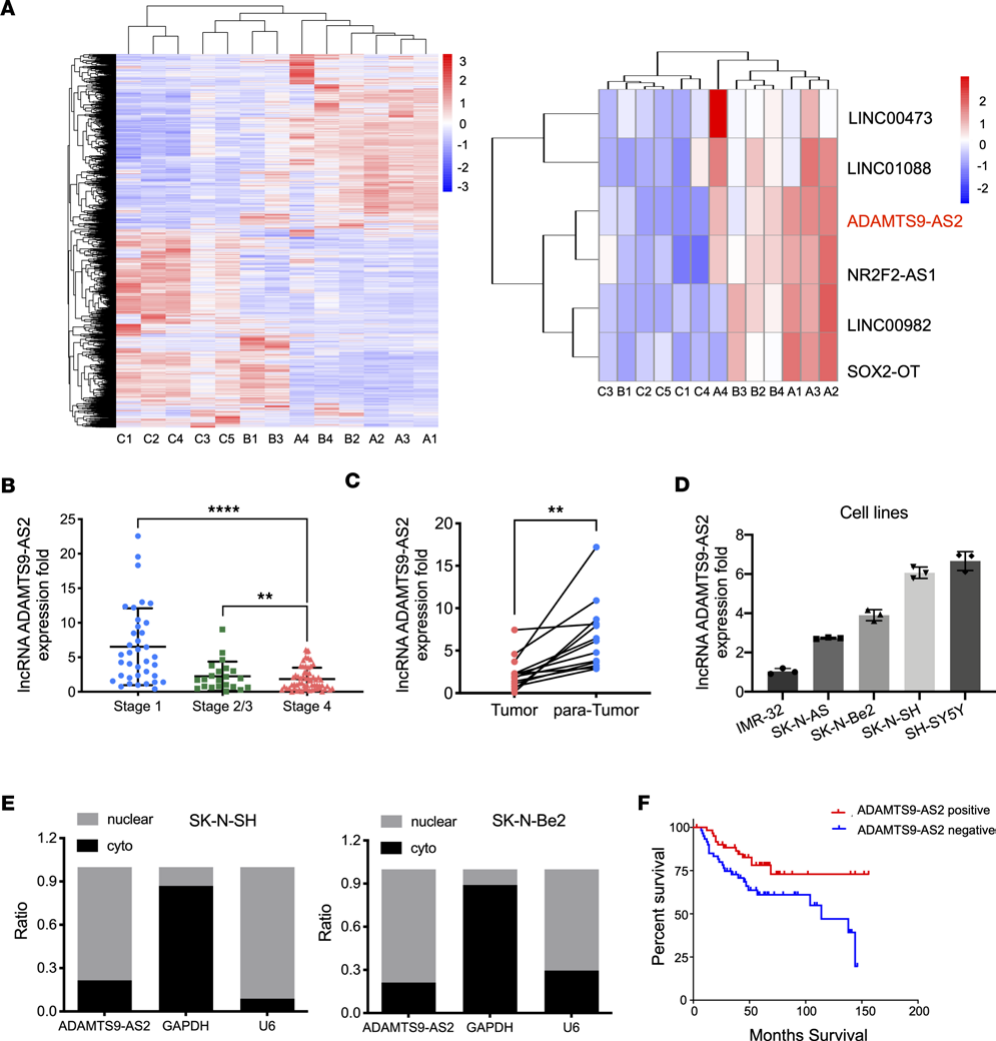
*ADAMTS9-AS2* is downregulated in high-risk human neuroblastoma tissues and cells. (**A**) RNA was extracted from high-risk and undifferentiated neuroblastoma (group C, *n* = 5), low-risk and differentiating ganglioneuroblastoma (group B, *n* = 4), and ganglioneuroma maturing subtype (group A, *n* = 4) samples. Left: Heatmap showing the clustering of samples based on mRNA and lncRNA expression. Right: Heatmap showing 6 lncRNAs that were most differentially expressed among the 3 groups. (**B**) Relative *ADAMTS9-AS2* expression in neuroblastoma samples from a large cohort (*n* = 121) of patients at different stages of the disease. (**C**) Real-time qRT-PCR assay was used to determine the relative expression of *ADAMTS9-AS2* in neuroblastoma and paired normal tissues (*n* = 13). (**D**) QRT-PCR analysis of *ADAMTS9-AS2* expression was conducted on 5 neuroblastoma cell lines (SK-N-Be2, SK-N-AS, SK-N-SH, SH-SY5Y, and IMR-32). Experiments were conducted in triplicate and data are presented as mean ± standard deviation (SD). (**E**) QRT-PCR was used to analyze *ADAMTS9-AS2* levels in nuclear and cytoplasmic fractions from SK-N-SH and SK-N-Be2 cells. GAPDH and U6 were used as cytoplasmic and nuclear control RNAs, respectively. (**F**) Kaplan-Meier correlation analysis was conducted on 121 patients with neuroblastoma to assess the relationship between *ADAMTS9-AS2* expression levels and overall survival (*n* = 121, stratified based on median expression of *ADAMTS9-AS2*, *P* = 0.0178). Data are presented as mean ± SD; statistical differences were calculated using unpaired 2-tailed Student’s *t* test was used. **P < 0.01, ****P < 0.0001.

We used high-throughput transcriptomic profiling to perform a comparative analysis of lncRNAs in neuroblastoma (high-risk and undifferentiated, *n* = 5), intermixed ganglioneuroblastoma (low-risk and differentiating, *n* = 4), and ganglioneuroma maturing subtype (*n* = 4) samples (Supplemental Table 1; supplemental material available online with this article; https://doi.org/10.1172/jci.insight.165703DS1). These comparisons revealed several differentially expressed transcripts, and our analysis of lowly differentiating and highly differentiating tumors identified several lncRNAs as top ranked candidates (Figure 1A). Quantitative real-time polymerase chain reaction (qRT-PCR) further verified a reduction in the expression levels of the candidate lncRNAs, including lncRNA *ADAMTS9-AS2*, in the 4 intermixed ganglioneuroblastoma and 5 neuroblastoma samples compared with the 4 ganglioneuroma maturing subtype samples (Supplemental Figure 1A). To investigate the expression of candidate lncRNAs in differentiating and undifferentiated neuroblastoma cells, we treated SH-SY5Y and SK-N-SH cells with retinoic acid for 7 days, differentiating neuroblastoma cells into neuronal like cells (Supplemental Figure 1, B and C). Analysis of candidate lncRNA expression over the course of neuronal differentiation showed upregulated *ADAMTS9-AS2* expression, with *ADAMTS9-AS2* being one of the top ranked candidates (Supplemental Figure 1D). We subsequently measured the expression levels of *ADAMTS9-AS2* in clinical neuroblastoma samples, and the results showed that the expression of *ADAMTS9-AS2* was markedly lower in patients with stage 4 tumors compared with in patients with stage 1 or stage 2 and 3 tumors (Figure 1B). Additionally, we used qRT-PCR to analyze *ADAMTS9-AS2* expression in tumor tissues and their paired para-tumor tissues in 13 paired neuroblastoma samples. *ADAMTS9-AS2* expression was significantly downregulated in neuroblastoma samples compared with the corresponding adjacent benign samples (Figure 1C). In Figure 1D, we also analyzed *ADAMTS9-AS2* expression in 5 neuroblastoma cell lines; the nonamplified cells (SH-SY5Y and SK-N-SH) had higher expression than the MYCN-amplified lines (SK-N-BE-2 and IMR-32). Analysis of nuclear and cytoplasmic fractionations of SK-N-Be2 and SK-N-SH cell lysates showed that *ADAMTS9-AS2* expression was higher in the nuclear fraction than in the cytoplasmic fraction (Figure 1E).

In addition, neuroblastoma samples with high *ADAMTS9-AS2* expression showed higher differentiation compared with neuroblastoma samples with low *ADAMTS9-AS2* expression (stratified based on median expression of *ADAMTS9-AS2* in 121 neuroblastoma samples, Supplemental Table 2, *P* = 0.016). *ADAMTS9-AS2* expression level was also closely correlated with the primary site (*P* = 0.044) but was negatively correlated with metastasis (*P* = 0.011) and clinical stage (*P* = 0.013). Kaplan-Meier analysis showed that patients with neuroblastoma who had low *ADAMTS9-AS2* levels had shorter overall survival (*P* = 0.0178; Figure 1F). Moreover, we analyzed the publicly available RNA-Seq neuroblastoma data set consisting of 498 human neuroblastoma samples, and Kaplan-Meier analysis showed that patients who had higher *ADAMTS9-AS2* levels had longer overall survival (*P* = 0.036; Supplemental Figure 1E). In general, these data illustrate that *ADAMTS9-AS2* expression is positively correlated with pathological differentiation, while lower *ADAMTS9-AS2* expression is associated with a more aggressive neuroblastoma phenotype.

### ADAMTS9-AS2 is pivotal for differentiation of neuroblastoma cells.

**Figure 2:**
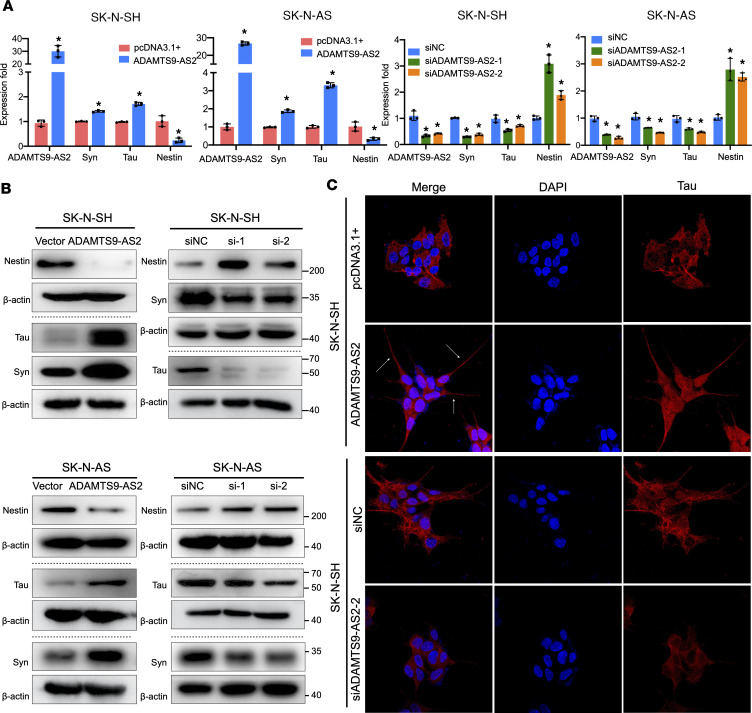
*ADAMTS9-AS2* expression is critical for neuronal differentiation of neuroblastoma cells. (**A** and **B**) SK-N-AS and SK-N-SH cells were transfected with control pcDNA3.1+ or *ADAMTS9-AS2*–overexpressing plasmids or control siRNA, *ADAMTS9-AS2* siRNA-1, or *ADAMTS9-AS2* siRNA-2 for 48 hours. *ADAMTS9-AS2*, *Syn*, *Tau*, and *Nestin* RNA expression was examined using qRT-PCR, with 18S rRNA as a control (**A**). Nestin, tau, Syn, and β-actin protein expression was analyzed using immunoblotting (**B**). Horizontal dotted lines separate blots on different gels, and values at right are shown in kilodaltons. (**C**) Immunostaining of pcDNA3.1+ and *ADAMTS9-AS2* cells (top); immunostaining of SK-N-SH cells treated with siNC or si*ADAMTS9-AS2* (bottom), using specific antibodies against the neuronal marker tau. The original magnification of **C** is ×1,000. Data are presented as mean ± SD; statistical analysis was performed by 2-tailed unpaired Student’s *t* test or 1-way ANOVA. *n* = 3, *P < 0.05.

Having demonstrated a clinical association between *ADAMTS9-AS2* expression levels and pathological differentiation in patients with neuroblastoma, we sought functional verification of the role of *ADAMTS9-AS2* in neuronal differentiation of neuroblastoma cells. First, we overexpressed *ADAMTS9-AS2* in SK-N-AS, SK-N-SH, SK-N-Be2, and IMR-32 cell lines, and the results showed that *ADAMTS9-AS2* overexpression upregulated the expression of neuronal markers (synaptophysin and tau) and downregulated the expression of a marker associated with stem cell properties (nestin). Next, we used 2 siRNAs to suppress *ADAMTS9-AS2* expression. In line with our hypothesis, suppressing *ADAMTS9-AS2* expression repressed neuronal differentiation, with the cells showing lower synaptophysin (Syn) and tau expression and higher nestin expression (Figure 2, A and B, and Supplemental Figure 2). Additionally, *ADAMTS9-AS2* upregulation in SK-N-SH cells increased tau expression and extended neurites (a characteristic of neuronal differentiation), facilitating neuronal differentiation. However, *ADAMTS9-AS2* downregulation inhibited tau expression and impaired neurite outgrowth in SK-N-SH cells (Figure 2C). Taken together, these results further underscore the importance of *ADAMTS9-AS2* in differentiation.

### Upregulation of ADAMTS9-AS2 inhibits proliferation and metastatic potential in neuroblastoma cell lines.

**Figure 3:**
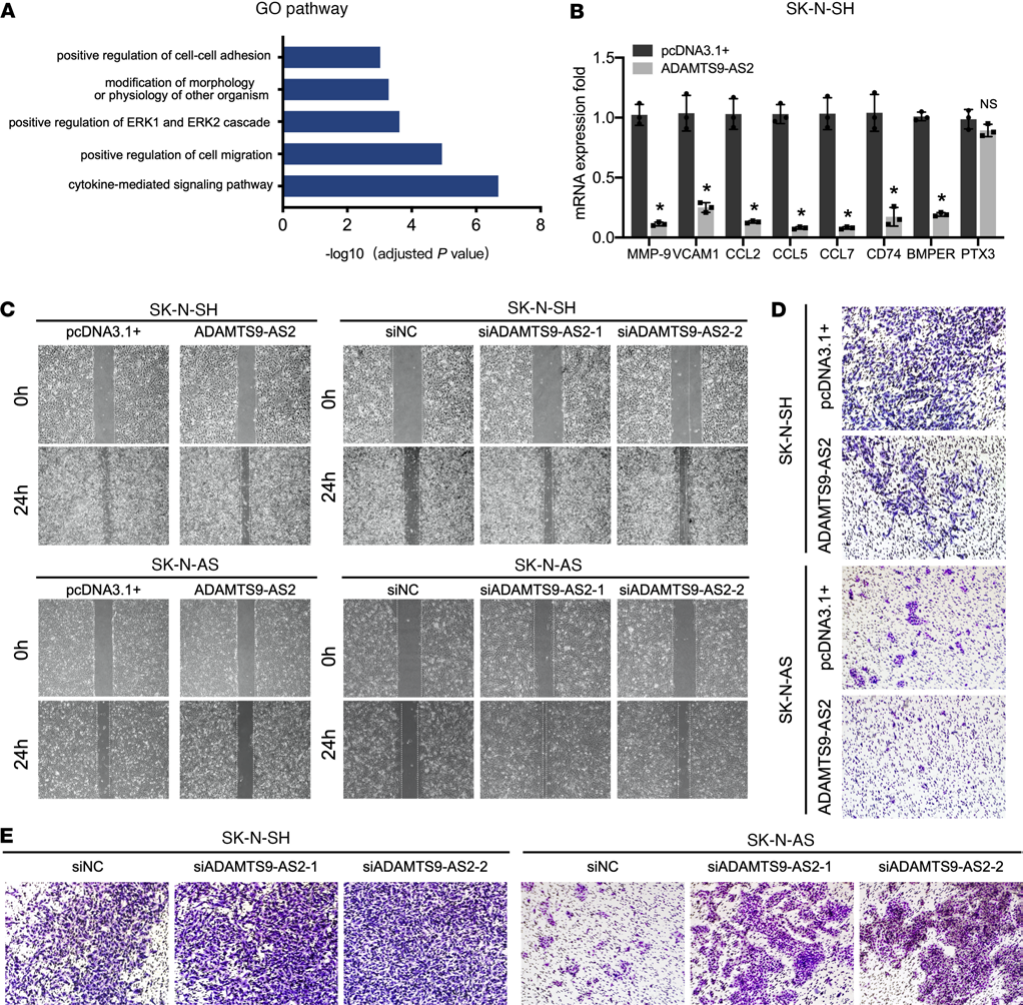
The lncRNA *ADAMTS9-AS2* harbors tumor-suppressive properties. (**A**) Gene Ontology analysis of the RNA-Seq data between control and *ADAMTS9-AS2*–overexpressing cells showing enrichment for biological processes associated with modification of morphology, regulation of ERK1 and ERK2 cascade, and cell migration. (**B**) QRT-PCR validation of some of the genes associated with cell migration and regulation of the ERK pathway in *ADAMTS9-AS2*–overexpressing cells (*n* = 3). Data are presented as mean ± SD; statistical analysis was performed by 2-tailed unpaired Student’s *t* test. *P < 0.05. (**C**) *ADAMTS9-AS2* upregulation in SK-N-SH and SK-N-AS cells impaired their migratory ability. SK-N-SH and SK-N-AS cells depleted of *ADAMTS9-AS2* had enhanced migratory capacity in wound-healing assays (*t* = 0 or 24 hours). (**D**) Invasion assays were performed using SK-N-AS and SK-N-SH cells transfected with pcDNA3.1+ and overexpressed *ADAMTS9-AS2*. (**E**) Invasion assays were conducted with SK-N-AS and SK-N-SH cells to detect the effects of *ADAMTS9-AS2* knockdown on cell invasion (original magnification, 100×).

To further explore the functional role of *ADAMTS9-AS2* in tumorigenesis, total RNA was extracted from *ADAMTS9-AS2*–overexpressing cells and vector control cells and sequenced. Gene Ontology (GO) analysis of *ADAMTS9-AS2*–overexpressing cells revealed an enrichment of genes associated with regulation of cell-cell adhesion and migration, modification of morphology, and regulation of the ERK1 and ERK2 cascade, indicating that *ADAMTS9-AS2* plays a critical role in migration and neuronal differentiation (Figure 3A and Supplemental Table 5). We verified some of the key genes associated with migration and ERK1/2 activation using qRT-PCR (Figure 3B). Next, we investigated the biological function of *ADAMTS9-AS2* in SK-N-AS and SK-N-SH cells using wound-healing and invasion assays. The results showed that upregulation of *ADAMTS9-AS2* reduced neuroblastoma cell metastasis. Consistent with data from *ADAMTS9-AS2*–overexpressing cells, transfection with *ADAMTS9-AS2* siRNAs promoted neuroblastoma cell migration and invasion (Figure 3, C–E, and Supplemental Figure 3, A and B). Moreover, depleting *ADAMTS9-AS2* significantly enhanced neuroblastoma cell proliferation, while *ADAMTS9-AS2* upregulation inhibited cell proliferation, as evidenced by cell viability and growth assays (Supplemental Figure 3, C and D). In summary, upregulated *ADAMTS9-AS2* expression inhibited the migration, invasion, and proliferation capabilities of neuroblastoma cells.

### The lncRNA ADAMTS9-AS2 interacts with LIN28B to inhibit the association between LIN28B and pri-let-7 in neuroblastoma cell lines.

**Figure 4:**
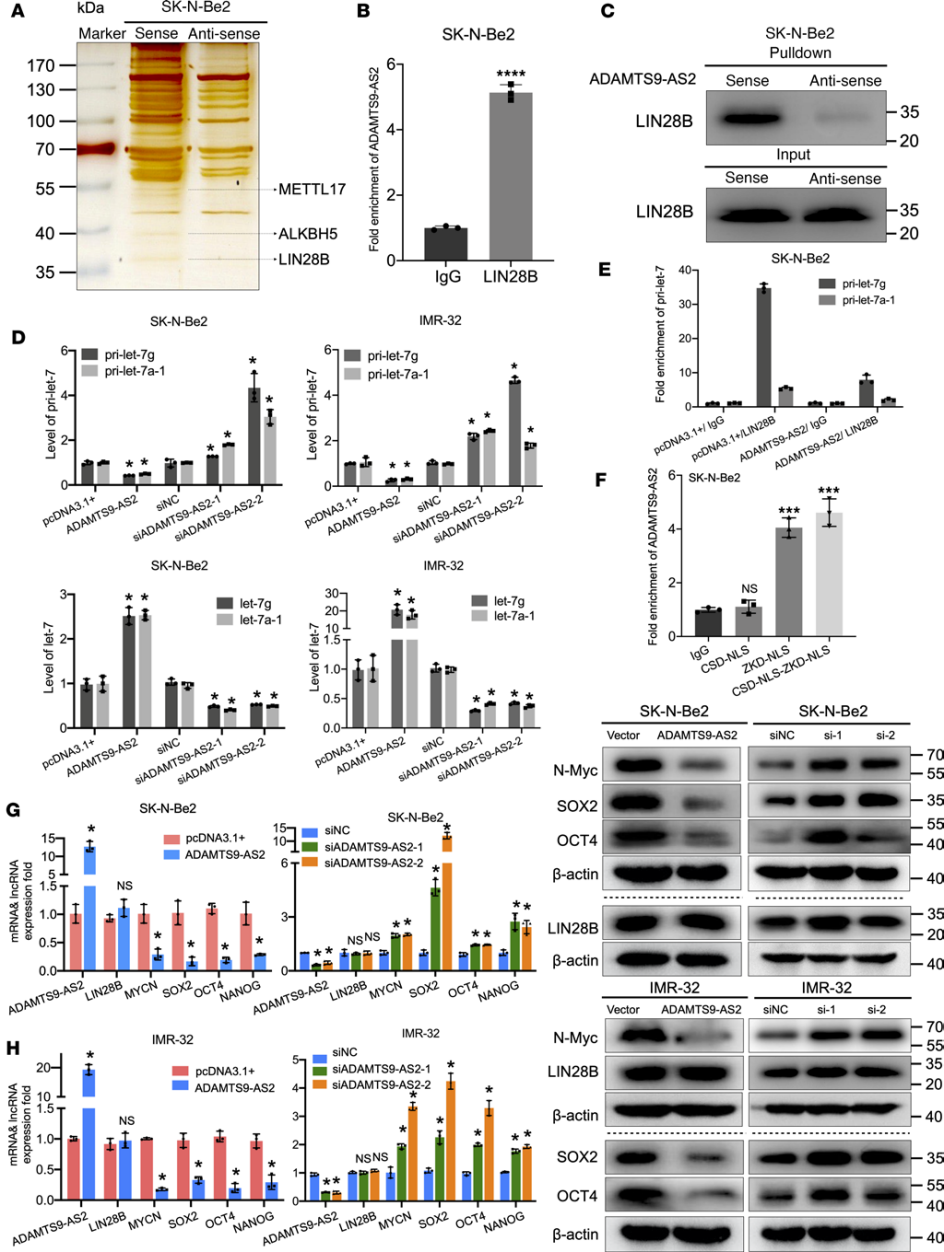
The lncRNA *ADAMTS9-AS2* interacts with LIN28B to inhibit the association between LIN28B and *pri-let-7*. (**A**) Image of an SDS-PAGE gel with silver staining. RNA pulldown was performed using SK-N-Be2 cell lysates. Biotinylated anti-sense probe was used as a control. (**B**) RIP enrichment was determined by comparing the relative levels of *ADAMTS9-AS2* in the immunoprecipitated LIN28B with the IgG control, with 18S rRNA serving as an internal standard (*n* = 3). (**C**) Biotinylated *ADAMTS9-AS2* was incubated with SK-N-Be2 cell lysate and then isolated using streptavidin-conjugated beads. Western blotting was conducted to evaluate the expression of LIN28B in both cell lysate input and RNA pulldown; biotinylated anti-sense *ADAMTS9-AS2* was used as the control. (**D**) *Pri-let-7* expression levels decreased and mature *let-7* accumulated following transient *ADAMTS9-AS2* expression. Accumulation of *pri-let-7* and decrease in *let-7g* expression using transient si*ADAMTS9-AS2* in IMR-32 and SK-N-Be2 cells detected by qRT-PCR (*n* = 3). (**E**) RIP was performed to examine the relative levels of *pri-let-7g* and *pri-let-7a-1* in the immunoprecipitates of LIN28B and compared with IgG control in the transient *ADAMTS9-AS2* and pcDNA3.1+ cells (*n* = 3). (**F**) Binding of *ADAMTS9-AS2* to Flag-tagged CSD-NLS, ZKD-NLS, and CSD-NLS-ZKD-NLS as determined using the RIP assay (*n* = 3). (**G** and **H**) ADAMTS9-AS2 was knocked down or overexpressed in SK-N-Be2 and IMR-32 cells. The levels of *ADAMTS9-AS2*, LIN28B, MYCN, and pluripotency transcription factors SOX2, OCT4, and NANOG were detected using qRT-PCR (*n* = 3). Immunoblots of MYCN, SOX2, OCT4, LIN28B, and β-actin were examined. Horizontal dotted lines separate blots on different gels. Experiments were conducted in triplicate and data are presented as mean ± SD. Statistical differences were calculated using unpaired 2-sided Student’s *t* test except for multiple-group comparisons for which 1-way ANOVA was used. *P < 0.05; ***P < 0.001; ****P < 0.0001.

LncRNAs interact with RNAs and proteins to regulate target genes and exert their functions. We used RNA pulldown assays followed by silver staining and mass spectrometry to detect proteins interacting with *ADAMTS9-AS2*. As shown in Figure 4A and Supplemental Figure 4A, *ADAMTS9-AS2* interacted with LIN28B, which is a potentially important candidate protein as it is known to bind RNA and is highly expressed in stem cells (24). The interaction between *ADAMTS9-AS2* and LIN28B was further verified using RNA immunoprecipitation (RIP) assay (Figure 4B). RNA pulldown assays identified LIN28B in the biotin-labeled *ADAMTS9-AS2* sense group but not in the antisense control group (Figure 4C). A previous study reported that LIN28B can directly bind to *pri-let-7*, preventing its cleavage by the microprocessor and selectively restraining *let-7* maturation (25). To understand the relationship between *ADAMTS9-AS2* and *let-7* maturation, we used qRT-PCR to measure the expression level of *pri-let-7*. *Pri-let-7* expression was downregulated by *ADAMTS9-AS2* overexpression and upregulated by *ADAMTS9-AS2* downregulation. Moreover, results showed a corresponding increase in mature *let-7* in *ADAMTS9-AS2*–overexpressing cells and remarkable decrease in mature *let-7* in *ADAMTS9-AS2*–knockdown cells (Figure 4D). We used RIP analysis to further verify that *ADAMTS9-AS2* bound LIN28B, preventing its binding to *pri-let-7* and promoting *let-7* maturation. *ADAMTS9-AS2* upregulation impaired the direct interaction between LIN28B and *pri-let-7*, suggesting that *ADAMTS9-AS2* can compete with *pri-let-7* for binding to LIN28B (Figure 4E). LIN28B has 2 major functional domains: zinc-knuckle domain (ZKD) and cold-shock domain (CSD). ZKD is critical for LIN28B binding to structurally diverse RNAs, such as *pri-let-7* (26, 27). To identify the LIN28B domains that are associated with *ADAMTS9-AS2*, we generated the major functional domain fragments of LIN28B. The results showed that *ADAMTS9-AS2* interacts with LIN28B through the ZKD domain (Figure 4F).

Previous studies have shown that LIN28B promotes MYCN expression by interacting with *pri-let-7* to inhibit *let-7* maturation, and increasing N-Myc protein levels was associated with MYCN amplification, which plays a crucial role in the tumorigenesis of neuroblastoma (28). Moreover, the LIN28B/*let-7* axis has been implicated in cancer cell stem-like property acquisition and regulation of key cancer stemness transcription factors (29). We investigated whether the association between *ADAMTS9-AS2* and LIN28B can regulate the expression of MYCN and stemness-related transcription factors. From the results in Figure 4, G and H, we could observe that *ADAMTS9-AS2* overexpression decreased MYCN, SOX2, OCT4, and NANOG mRNA and protein expression in both SK-N-Be2 and IMR-32 cells. Knocking down *ADAMTS9-AS2* with siRNA elevated the mRNA and protein expression levels of MYCN and pluripotency transcription factors. However, *ADAMTS9-AS2* expression levels had no influence on LIN28B expression. To ascertain the direct impact of *ADAMTS9-AS2* via LIN28B/*let-7* on MYCN expression at the posttranscriptional level, we examined expression of MYCN using the plasmid of MYCN and intact 3′UTR and MYCN and *let-7* site mutant 3′UTR in non–MYCN-amplified SK-N-SH cells. The expression of MYCN was inhibited when *ADAMTS9-AS2* was highly expressed, but the expression of MYCN was restored when the *let-7* binding site was mutated in SK-N-SH cells (Supplemental Figure 4B). Overall, these results indicate that *ADAMTS9-AS2* regulates MYCN expression at the posttranscriptional level by binding to LIN28B.

### ADAMTS9-AS2 regulates neuroblastoma stem-like properties via LIN28B/let-7 signaling.

**Figure 5:**
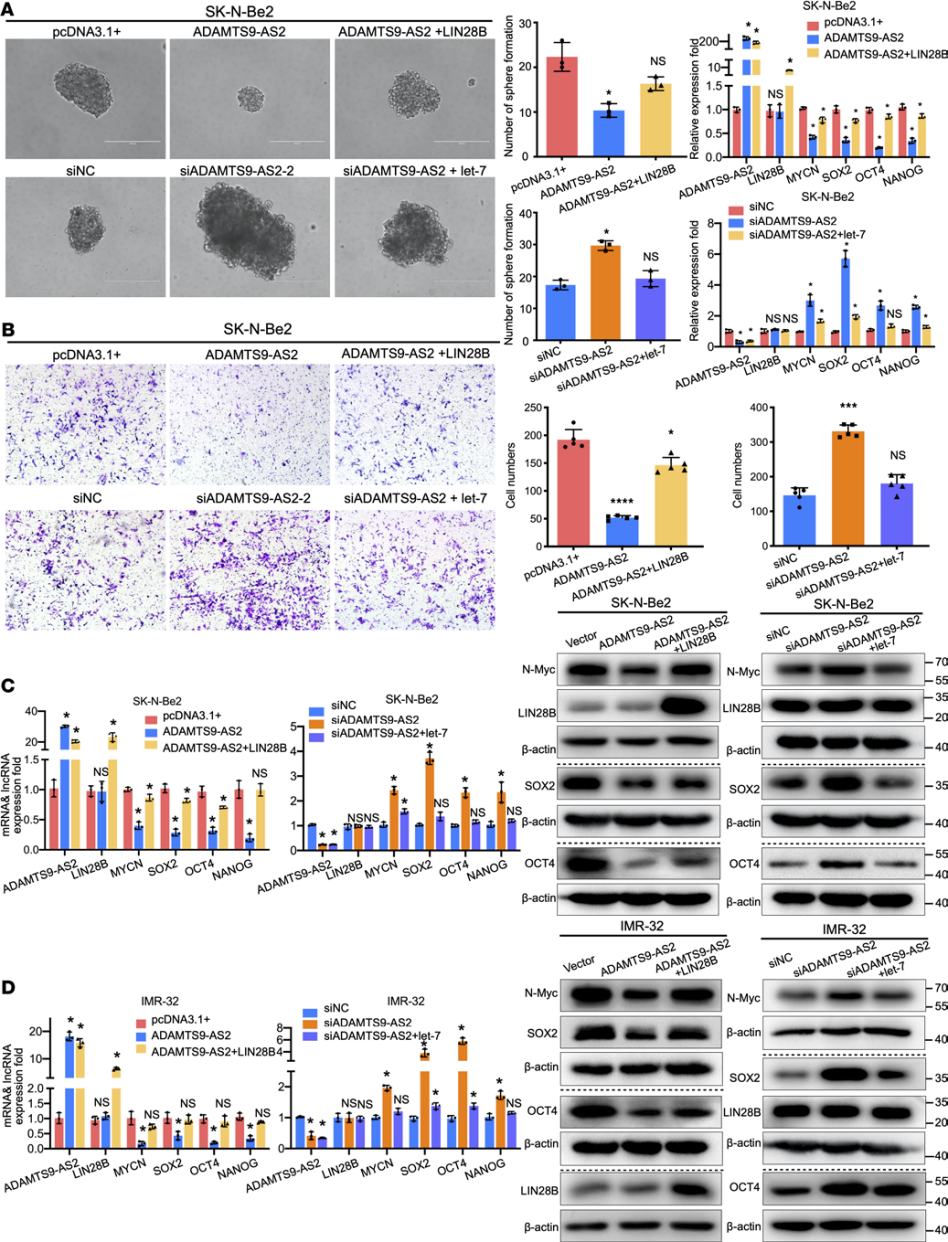
*ADAMTS9-AS2* regulates neuroblastoma stem-like properties through LIN28B/*let-7* signaling. (**A** and **B**) LIN28B was overexpressed in SK-N-Be2/*ADAMTS9-AS2* cells. *Let-7* was overexpressed in SK-N-Be2/si*ADAMTS9-AS2* cells. Spheroid formation (*n* = 3) and invasion assays were performed (*n* = 5). A representative image and histogram are shown. QRT-PCR was used to examine relative mRNA expression of *LIN28B*, *MYCN*, *SOX2*, *OCT4*, and *NANOG* (*n* = 3). The scale bar in **A** is 200 μm. The original magnification of **B** is 100×. (**C**) QRT-PCR analysis of *ADAMTS9-AS2*, *LIN28B*, *MYCN*, *SOX2*, *OCT4*, and *NANOG* levels (left, *n* = 3); MYCN, SOX2, OCT4, LIN28B, and β-actin levels were measured in pcDNA3.1+, *ADAMTS9-AS2*, and *ADAMTS9-AS2*+LIN28B cells using immunoblots (right). (**D**) IMR-32 and SK-N-Be2 cells transfected with siNC, si*ADAMTS9-AS2*, and si*ADAMTS9-AS2*+*let-7* were subjected to qRT-PCR analysis of *ADAMTS9-AS2*, *LIN28B*, *MYCN*, *SOX2*, *OCT4*, and *NANOG* expression (left, *n* = 3) and Western blot analysis for MYCN, SOX2, OCT4, LIN28B, and β-actin levels (right). Horizontal dotted lines separate blots on different gels. Data are presented as mean ± SD. Statistical differences were calculated using unpaired 2-sided Student’s *t* test except for multiple-group comparisons for which 1-way ANOVA was used. *P < 0.05; ***P < 0.001; ****P < 0.0001.

LIN28B/*let-7* signaling mediates stemness acquisition and induces cancer stem-like properties and tumorigenesis (29). Therefore, we investigated the role of *ADAMTS9-AS2* in neuroblastoma cellular stemness. We found that *ADAMTS9-AS2* overexpression inhibited cancer stem-like cell sphere formation capability, while LIN28B upregulation rescued the neuroblastoma stem-like properties. On the contrary, *ADAMTS9-AS2* downregulation induced the formation of larger spheres containing more cells compared with controls, while *let-7* overexpression blocked the cancer stemness ability (Figure 5A). Moreover, the reduction in mRNA levels of *MYCN* and stemness-related transcription factors in cancer stem-like cells that was impaired by upregulated *ADAMTS9-AS2* expression was rescued by overexpressing LIN28B. The increase in *MYCN*, *SOX2*, *OCT4*, and *NANOG* mRNA expression induced by inhibiting *ADAMTS9-AS2* expression was rescued by overexpressing *let-7*. These results further illustrate that *ADAMTS9-AS2* regulates stem cell states through LIN28B/*let-7* signaling. Cancer stem cells (CSCs) are the seed for metastasis and the driving force for tumorigenesis (30). To verify whether *ADAMTS9-AS2* inhibits metastasis through the LIN28B/*let-7* pathway, we first stimulated LIN28B expression in *ADAMTS9-AS2*–overexpressing cells and upregulated *let-7* expression in *ADAMTS9-AS2*–knockdown cells. Re-executed invasion assay demonstrated that LIN28B could stimulate *ADAMTS9-AS2* overexpression–induced inhibition of metastasis, and *let-7* could attenuate *ADAMTS9-AS2* knockdown–induced promotion of metastasis (Figure 5B).

We next investigated whether LIN28B or *let-7* upregulation in MYCN-amplifying SK-N-Be2 and IMR-32 cells affects *ADAMTS9-AS2* and its downstream genes. The results indicated that LIN28B and *let-7* were indeed located downstream of *ADAMTS9-AS2*. We also verified that upregulating LIN28B expression restored the mRNA and protein expression levels of MYCN and stemness-related transcription factors (SOX2 and OCT4) in SK-N-Be2 and IMR-32 cells overexpressing *ADAMTS9-AS2*. Conversely, *let-7* upregulation partly reversed the enhanced MYCN, SOX2, and OCT4 expression effect of *ADAMTS9-AS2* downregulation (Figure 5, C and D).

Furthermore, we observed an inverse correlation between *ADAMTS9-AS2* and *MYCN* and a positive correlation between *ADAMTS9-AS2* and *let-7a-1* in an analysis of clinical samples (*n* = 33 cases, Supplemental Figure 5A). Analysis of the R2 Genomics Analysis and Visualization Platform (http://r2.amc.nl) further verified that *ADAMTS9-AS2* expression was negatively correlated with *MYCN* in patients with neuroblastoma (Pearson’s correlation coefficient –0.360, *P* < 0.0001, Supplemental Figure 5B). Although *ADAMTS9-AS2* is important for the regulation of MYCN, the expression of *ADAMTS9-AS2* was no different in MYCN-amplified and nonamplified samples (Supplemental Figure 5C). Taken together, our results support the hypothesis that *ADAMTS9-AS2* regulates neuroblastoma stem-like properties and MYCN expression through LIN28B/*let-7* signaling. *ADAMTS9-AS2* impaired LIN28B/*let-7* mediated stem-like and metastasis properties via directly binding to LIN28B in neuroblastoma cells.

### N^6^-methyladenosine modification regulates the expression of ADAMTS9-AS2 in neuroblastoma cells.

**Figure 6:**
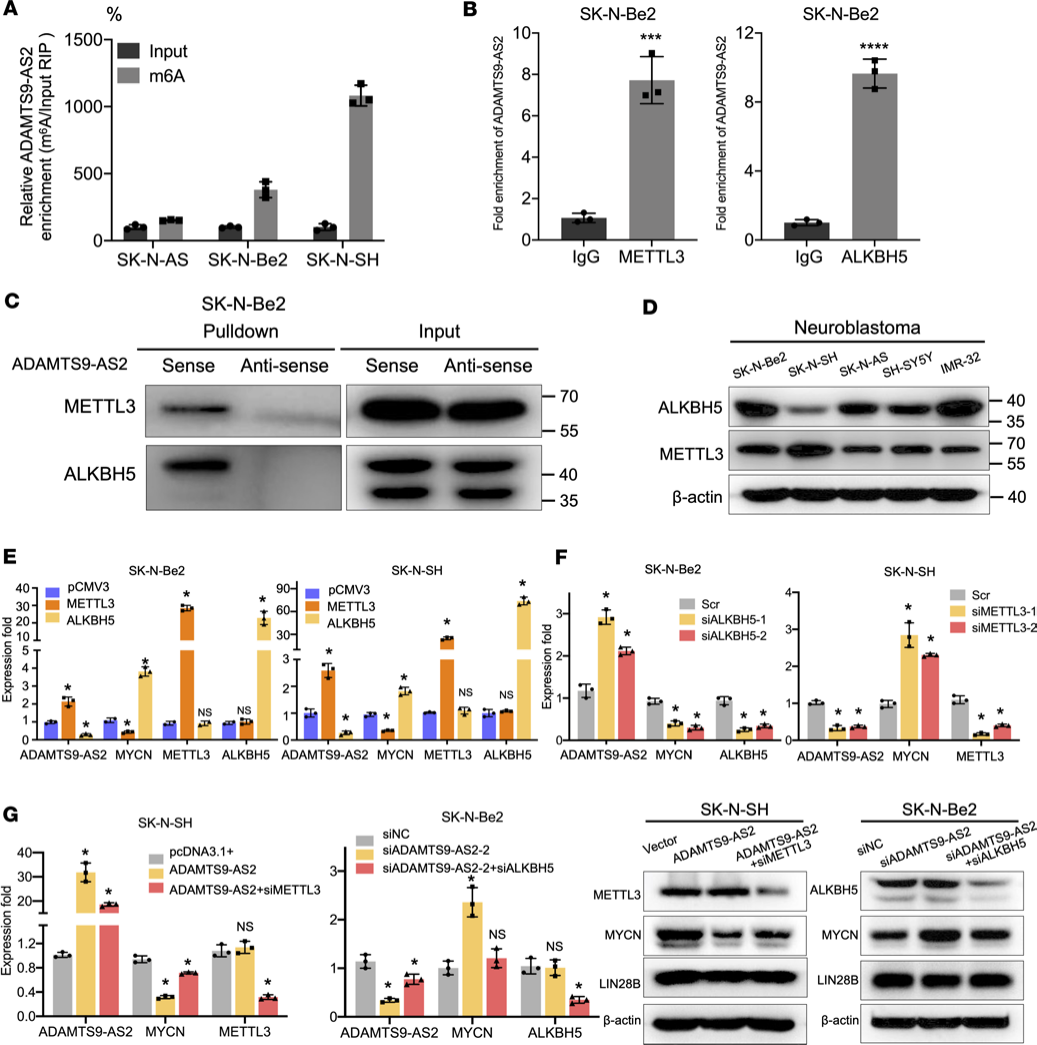
*ADAMTS9-AS2* expression levels in neuroblastoma cells is regulated by m^6^A modification. (**A**) M^6^A RIP and qRT-PCR analyses were conducted in neuroblastoma cells (*n* = 3). (**B**) Graphs showing enrichment of *ADAMTS9-AS2* in the METTL3 or ALKBH5 immunoprecipitated RNA fraction of SK-N-Be2 cells (*n* = 3). (**C**) RNA pulldown assay showed the interaction between *ADAMTS9-AS2* and METTL3/ALKBH5. (**D**) METTL3 and ALKBH5 expression in 5 neuroblastoma cell lines. (**E**) *ADAMTS9-AS2* and MYCN expression levels were evaluated in neuroblastoma cells overexpressing METTL3 or ALKBH5 (*n* = 3). (**F**) The expression level of *ADAMTS9-AS2* and MYCN was assessed in SK-N-Be2 cells with ALKBH5 knockdown and SK-N-SH cells with METTL3 knockdown (*n* = 3). (**G**) pcDNA3.1+-, *ADAMTS9-AS2*–, and *ADAMTS9-AS2*+siMETTL3–transfected SK-N-SH cells and siNC-, si*ADAMTS9-AS2*–, and si*ADAMTS9-AS2*+siALKBH5–transfected SK-N-Be2 cells were subjected to measurement of *ADAMTS9-AS2* and MYCN expression. Experiments were conducted in triplicate and data are presented as mean ± SD. Statistical differences were calculated using unpaired 2-tailed Student’s *t* test except for multiple-group comparisons for which 1-way ANOVA was used. *P < 0.05; ***P < 0.001; ****P < 0.0001.

Recent research into tumor epigenetic regulation has shed light on the role of N^6^-methyladenosine (m^6^A) posttranscriptional modification in the regulation of mRNA and lncRNA and shown that m^6^A affects mRNA and lncRNA stability (31, 32). Methyltransferase-like 17 (METTL17) is an RNA methyltransferase, and AlkB homolog 5 (ALKBH5) is a key m^6^A demethylase. Our results showed that they both specifically interacted with *ADAMTS9-AS2* (Figure 4A and Supplemental Figure 4). Moreover, we identified multiple m^6^A binding motifs in *ADAMTS9-AS2* in the online bioinformatics database, m6Avar (33). We analyzed whether m^6^A modification is associated with the diverse expression of *ADAMTS9-AS2* in neuroblastoma. M^6^A RIP and qRT-PCR assay showed 1.5-fold, 3.8-fold, and 10.8-fold enrichment in m^6^A antibody levels of *ADAMTS9-AS2* in SK-N-AS, SK-N-Be2, and SK-N-SH cells, respectively (Figure 6A), potentially accounting for the differences in the *ADAMTS9-AS2* expression observed in these cell lines (Figure 1D). We also validated METTL3 and ALKBH5 binding to *ADAMTS9-AS2* in neuroblastoma cells using RIP followed by qRT-PCR and pulldown assays coupled with Western blotting (Figure 6, B and C). The endogenous expressions of METTL3 and ALKBH5 in the neuroblastoma cell lines were analyzed; the results showed that ALKBH5 expression was lowest and that METTL3 expression was highest in the SK-N-SH cells (Figure 6D). Furthermore, we found that upregulating METTL3, the crucial m^6^A methyltransferase (34), increased *ADAMTS9-AS2* expression and decreased MYCN expression in SK-N-Be2 and SK-N-SH cells. Consistently, ALKBH5 upregulation impaired *ADAMTS9-AS2* expression and increased MYCN expression (Figure 6E and Supplemental Figure 6A). To explore the effects of METTL3 and ALKBH5 on *ADAMTS9-AS2* upregulation in neuroblastoma cells, we knocked down the expression of METTL3 in SK-N-SH cells and knocked down ALKBH5 in SK-N-Be2 cells. We found that METTL3 downregulation was associated with decreased *ADAMTS9-AS2* and increased MYCN expression level. On the other hand, ALKBH5 downregulation was associated with upregulated *ADAMTS9-AS2* and inhibited MYCN expression level (Figure 6F and Supplemental Figure 6B). We also verified that downregulating ALKBH5 expression restored *ADAMTS9-AS2* expression and impaired MYCN expression in *ADAMTS9-AS2*–knockdown cells. Conversely, METTL3 downregulation decreased *ADAMTS9-AS2* expression and partly reversed the decreased MYCN expression effect of *ADAMTS9-AS2* overexpression (Figure 6G). These results demonstrate that m^6^A positively modulates *ADAMTS9-AS2* expression and impairs its downstream MYCN expression in neuroblastoma cells.

### ADAMTS9-AS2 upregulation inhibits proliferation and cancer stem-like capabilities in vivo.

**Figure 7:**
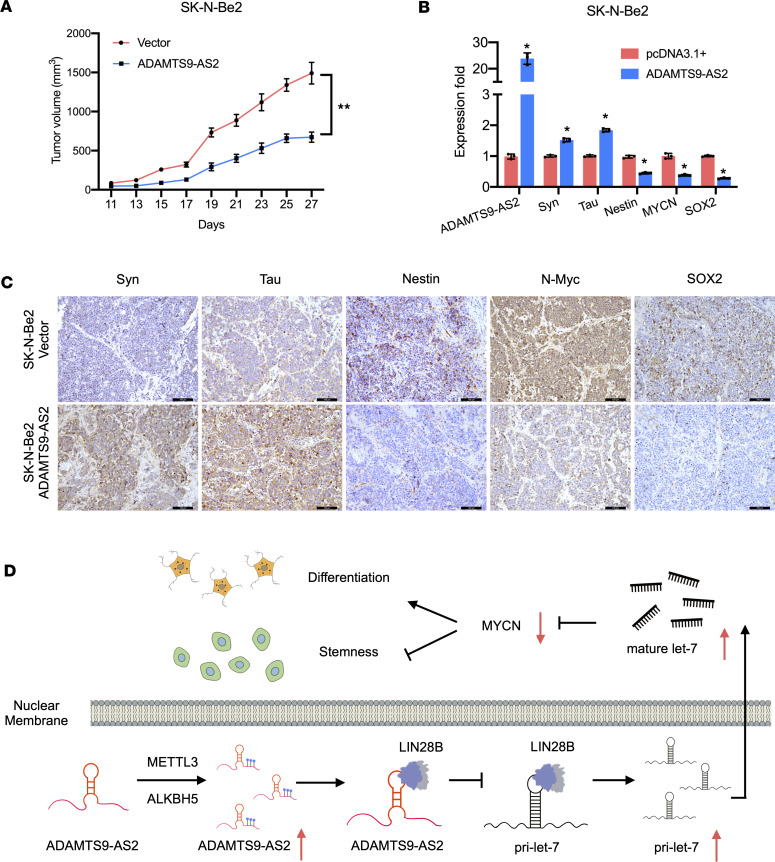
*ADAMTS9-AS2* inhibits proliferation and cancer stem-like capabilities in vivo. (**A**) Tumor volume was measured (*n* = 5). (**B** and **C**) Syn, tau, nestin, MYCN, and SOX2 expression levels were determined in *ADAMTS9-AS2*–overexpressing SK-N-Be2 cells and control tumor tissues using qRT-PCR and immunohistochemistry (original magnification, 200×) assays. (**D**) Schematic representation of the function of *ADAMTS9-AS2* in the LIN28B/*let-7*/MYCN axis and its role in suppressing cancer stem cell properties and inducing neuroblastoma cell differentiation. Data are presented as mean ± SD; statistical differences were calculated using unpaired 2-tailed Student’s *t* test. *P < 0.05; **P < 0.01.

To explore the capability of *ADAMTS9-AS2* in vivo, we used a xenograft model to verify the influence of *ADAMTS9-AS2* on proliferation, differentiation, and stem-like capabilities of neuroblastoma cells. Subcutaneous tumor formation experiments revealed that adenovirus-mediated overexpression of *ADAMTS9-AS2* suppressed tumor formation (Figure 7A). qRT-PCR analysis of xenograft tumor samples showed that the *ADAMTS9-AS2*–overexpressing group had higher levels of differentiation markers (*Syn* and *Tau*) and lower levels of *MYCN* and stem cell markers (*Nestin* and *SOX2*) compared with SK-N-Be2 cell xenograft vehicle groups (Figure 7B). Furthermore, histochemistry staining of histological sections clearly showed that *ADAMTS9-AS2* overexpression markedly accelerated Syn and tau expression, whereas *ADAMTS9-AS2* upregulation remarkably inhibited nestin, N-Myc, and SOX2 expression (Figure 7C). Together, these findings demonstrate that *ADAMTS9-AS2* overexpression inhibits proliferation and cancer stem-like capabilities in vivo.

## Discussion

Neuroblastomas frequently transdifferentiate into more benign ganglioneuroblastomas and ganglioneuromas. Ganglioneuroblastoma is a member of peripheral neuroblastic tumors. Human neuroblastoma cells are induced to differentiate into cells possessing properties characteristic of mature ganglion cells, dividing ganglioneuroblastoma into 2 types of tumor cells: undifferentiated neuroblastoma cells and mature ganglion cells, which are interconvertible (35). This makes it the best model for studying cell differentiation. In this study, we identified differentially expressed lncRNAs in neuroblastoma, ganglioneuroblastoma, and ganglioneuroma and then characterized the lncRNAs that can distinguish the degree of differentiation in neuroblastomas. Retinoic acid has been used with some success as a maintenance therapy for high-risk neuroblastoma, and treating SH-SY5Y cells with retinoic acid gave the cells the ability to differentiate into neurons (36). This neuroblastoma cell differentiation model and clinical samples from different clinical stages and pathological differentiation were used to study the candidate lncRNAs. The analysis identified *ADAMTS9-AS2* as one of the top ranked candidates. *ADAMTS9-AS2* was negatively associated with advanced stage of neuroblastoma and poor outcome. *ADAMTS9-AS2* upregulation enhanced cell differentiation and decreased cell proliferation, metastasis, and cancer stem-like properties, and *ADAMTS9-AS2* downregulation promoted malignant potential in neuroblastoma cells. Moreover, *ADAMTS9-AS2* overexpression led to neuronal differentiation by upregulating several key neuron-specific genes and consequent downregulation of the stemness marker. These observations indicate that *ADAMTS9-AS2* may be a critical lncRNA affecting neuronal fate commitment.

MYCN, a member of the Myc family that encodes transcription factors, plays a critical role in neuroblastoma tumorigenesis, including promoting cell growth, differentiation, and development of aggressive phenotypes. Abnormal MYCN amplification and the consequent overexpression of the oncoprotein, N-Myc, which occurs in 20%–25% of patients with neuroblastoma and 50% of high-risk cases, is the most important promoting factor and the best-characterized genetic marker for poor prognosis of neuroblastoma (37–39). Over the last decades, several genes have been demonstrated to enhance or weaken the tumorigenic effect of N-Myc. LIN28B, a neuroblastoma susceptibility gene that is involved in disease initiation, promotes MYCN expression (40). LIN28B inhibits the maturation of the tumor suppressor *let-7* biogenesis, which regulates crucial biological functions such as promoting differentiation, restraining cell proliferation, and repressing MYCN expression (25, 41). We propose an essential regulatory role for *ADAMTS9-AS2* in neuroblastoma, influencing LIN28B/*let-7*/MYCN axis signaling. Highlighting the pivotal relationship between LIN28B and *let-7*, we showed that LIN28B is an *ADAMTS9-AS2* target. Even though *ADAMTS9-AS2* does not influence LIN28B expression, it can inhibit the binding between LIN28B and *pri-let-7* by competing for binding to the ZKD domain of LIN28B. Then *pri-let-7* is exported from the nucleus to the cytoplasm, where it forms mature *let-7*, which targets and suppresses the expression of MYCN. Although *ADAMTS9-AS2* is important for the regulation of MYCN, the expression of *ADAMTS9-AS2* was no different in MYCN-amplified and nonamplified samples. Our results showed the direct impact of *ADAMTS9-AS2* via LIN28B/*let-7* on MYCN expression at the posttranscriptional level. Accordingly, we speculate that *ADAMTS9-AS2*, through its interaction with LIN28B, is a key upstream component of the LIN28B/*let-7*/MYCN axis.

N-Myc and LIN28B enhance the production of induced pluripotent stem cells and play an important role in maintaining CSC properties (42–44). The LIN28 family, together with NANOG, OCT4, and SOX2, can induce stem cell self-renewal and reprogram human somatic cells into pluripotent stem cells. CSCs are considered major causes of tumor relapse, chemoresistance, radioresistance, and metastasis. Researchers have revealed that neuroblastoma contains a cell population with stem cell–like characteristics with increased expression of CSC markers, such as nestin, OCT4, CD133, and ALDH (45, 46). We therefore sought to comprehensively explore the effects of *ADAMTS9-AS2* on neuroblastoma CSCs and how this alters overall tumor growth and metastasis. *ADAMTS9-AS2* inhibited stem cell function and metastasis ability through the LIN28B/*let-7*/MYCN axis. Moreover, *ADAMTS9-AS2* regulates key cancer stemness transcription factors both in vitro and in vivo. Many recent studies have focused on targeting CSCs to eliminate malignancies by regulating stem cell pathways, and products based on these strategies are in preclinical and clinical trials (47). In this study, we hypothesized that *ADAMTS9-AS2* may be used as a new therapeutic strategy to target CSCs and promote cancer cell differentiation.

M^6^A modification controlled the fate of mRNA and stabilized methylated mRNA (47, 48). M^6^A deposition is encoded by “writers” (such as METTL3 and METTL14) that catalyze m^6^A formation, “erasers” (such as ALKBH5) that selectively remove the m^6^A code, and “readers” (such as IGF2BP and YTH domain proteins) that decode m^6^A methylation (49). M^6^A influences the targeted lncRNA and is involved in the progression of numerous cancers. Research demonstrated that m^6^A modification was highly enriched on LINC00958 in hepatocellular carcinoma and enhanced RNA stability in an METTL3-dependent manner (50). M^6^A modification also promoted lncRNA RP11 expression by enhancing its accumulation in the nuclei of colorectal cancer cells (51). In this study, our data first identified that m^6^A methylation was enriched in *ADAMTS9-AS2* using m^6^A RIP assay in SK-N-SH cells, which could account for *ADAMTS9-AS2* differential expression in neuroblastoma cells. Additionally, we found that METTL3 and ALKBH5 bound to *ADAMTS9-AS2* and the expression of METTL3 and ALKBH5 controlled *ADAMTS9-AS2* stability and expression in an m^6^A-dependent manner. These results indicate that *ADAMTS9-AS2* expression in neuroblastoma may be attributed to m^6^A modification. The endogenous expressions of METTL3 and ALKBH5 of the neuroblastoma cell lines regulated the m^6^A modification in *ADAMTS9-AS2*, thus affecting its RNA stability and its downstream MYCN expression. Since the mechanism underlying RNA methylation is still poorly understood, additional discoveries of the effects of regulatory patterns mediated by m^6^A on the functions and biogenesis of lncRNA, particularly *ADAMTS9-AS2*, should be verified in future studies.

In conclusion, we showed that the expression levels of the lncRNA *ADAMTS9-AS2* were positively associated with pathological differentiation and negatively correlated with aggressive neuroblastoma phenotype. The observed differential expression of *ADAMTS9-AS2* in neuroblastoma cells was caused by m^6^A methylation. Additionally, we verified that *ADAMTS9-AS2* upregulation in neuroblastoma cells promotes neuronal differentiation and inhibits cell proliferation and metastasis. Furthermore, we identified LIN28B as a key protein associated with *ADAMTS9-AS2* and showed that the interaction between *ADAMTS9-AS2* and LIN28B restrains the association between LIN28B and *pri-let-7*, leading to the release of *pri-let-7* into the cytoplasm to form mature *let-7*, which subsequently inhibits MYCN activity (Figure 7D). Elucidating the function of *ADAMTS9-AS2* in the LIN28B/*let-7*/MYCN axis and its role in suppressing proliferation, metastasis, and CSC properties and inducing neuroblastoma cell differentiation may open novel avenues for developing therapeutic approaches and pharmacological interventions.

## Methods

### Cell lines.

SK-N-AS, SH-SY5Y, SK-N-Be2, and HEK293T cell lines were obtained from American Type Culture Collection (USA), and SK-N-SH and IMR-32 cell lines were purchased from Type Culture Collection of the Chinese Academy of Sciences (China). All cell lines were routinely tested for mycoplasma. Basal medium (DMEM for SK-N-AS, SH-SY5Y, SK-N-SH, HEK293T; MEM for IMR-32; DMEM/Nutrient Mixture F-12 for SK-N-Be2) supplemented with 10% FBS and 1% penicillin/streptomycin was used to culture the cell lines. Cells were maintained at 5% CO_2_.

### Sample source.

A total of 134 neuroblastoma samples were collected and histopathologically diagnosed at the Tianjin Medical University Cancer Institute and Hospital (Tianjin, China) between 2008 and 2021 (Supplemental Tables 1 and 2). The samples were immediately snap-frozen in liquid nitrogen and stored at –80°C. Prior informed consent was obtained from each patient, and approval for the study was obtained from the Ethical Committee on Scientific Research of Tianjin Medical University Cancer Institute and Hospital (E20210664).

### Plasmids, siRNAs, and cell transfection.

Full-length complementary DNA (cDNA) of the lncRNA *ADAMTS9-AS2* was subcloned into the pcDNA3.1+ vector (Invitrogen). An empty pcDNA3.1+ was used as the negative control. Scrambled siRNA and siRNA targeting *ADAMTS9-AS2* were synthesized by GenePharma. The siRNA sequences used are listed in Supplemental Table 3. Lipofectamine 3000 transfection reagent (Invitrogen) was used to transfect the plasmid and siRNA into neuroblastoma cells following the manufacturer’s instructions.

### Antibodies.

Antibodies used for immunoblotting, immunohistochemistry, immunofluorescence, and immunoprecipitation included: anti-nestin (Santa Cruz Biotechnology, catalog sc-23927), anti–β-actin (Cell Signaling Technology, catalog 4967S), anti-synaptophysin (Abcam, catalog ab32127), anti-tau (Abcam, catalog ab76128), anti-LIN28B (Abcam, catalog ab191881), anti–N-Myc (D4B2Y, Cell Signaling Technology, catalog 51705S), anti-SOX2 (Cell Signaling Technology, catalog 3579), anti-OCT4 (Cell Signaling Technology, catalog 2750), anti-METTL3 (Abcam, catalog ab195352), anti-ALKBH5 (Cell Signaling Technology, catalog 80283S), and anti–m^6^A (Abcam, catalog ab208577).

### RNA extraction and qRT-PCR analysis.

TRIzol reagent (Invitrogen) was used to extract RNA from neuroblastoma cells and tissues. cDNA was synthesized using the PrimeScript RT reagent kit (TAKARA). TB Green Premix Ex Taq II (TAKARA) was used for fluorescence quantitation. Quantitative PCR was conducted on the Applied Biosystems 7500 Real-Time PCR System. The standard 2^-ΔΔCt^ algorithm was used to calculate relative RNA expression. Sequences of the primers used are detailed in Supplemental Table 4.

MiRNA was quantitatively analyzed using reverse transcription reaction and qRT-PCR. Hairpin-it miRNAs RT-PCR Quantitation Kit (GenePharma) was used for quantitative analysis of miRNA. Reverse transcription was performed according to the user manual, and the cDNA products were used for qRT-PCR according to the manufacturer’s protocol. U6 small nuclear RNA expression was used as a standardized reference.

### Cytoplasmic and nuclear RNA purification.

Cytoplasmic and nuclear RNA were isolated from neuroblastoma cells using the Cytoplasmic and Nuclear RNA Purification Kit (Norgen). Briefly, after 3 washes with PBS, Lysis Buffer J was added to the culture plate containing the neuroblastoma cells. The lysates were centrifuged into a supernatant and a pellet containing RNA derived from cytoplasm and nuclei. Ethanol and buffer SK solutions were added to the desired fraction, and the mixture was loaded onto a spin column. Bound RNA was purified by washing with Wash Solution A and eluted with Elution Buffer E. Purified RNA samples were used for qRT-PCR.

### RNA pulldown assay.

Streptavidin Dynabeads (Invitrogen) were washed using Wash Solution (Solution A: 0.1 M NaOH, 0.1 M NaCl; Solution B: 0.1 M NaCl) and added to binding buffer supplemented with tRNA and RNase inhibitor (Promega). Biotinylated sense and antisense *ADAMTS9-AS2* RNA was heated for 2 minutes at 90°C and then placed on ice for 2 minutes. After incubating in RNA structure buffer, the RNA sample was mixed with beads in binding buffer. After washing with PBS, SK-N-Be2 cells were lysed for 30 minutes at 4°C with Tris-Triton (40 mM Tris, 120 mM NaCl, 1% Triton X-100, Roche protease inhibitor cocktail, Promega RNase inhibitor). Cell lysates were centrifuged for 10 minutes at 12,000 rpm and then incubated overnight at 4°C with beads. Finally, the beads were washed in lysis buffer and then subjected to Western blot analysis. The pulldown assay was performed as described previously (52).

### RIP.

RIP was performed to research the interactions between proteins and RNA in cells as described previously (24). Protein A/G magnetic beads (Invitrogen) were incubated with antibodies or IgG at 4°C with rotation. SK-N-Be2 cells were lysed with Tris-Triton supplemented with protease and RNase inhibitor, and the cell lysates were mixed with beads at 4°C. The beads were then washed with lysis buffer. The co-precipitated RNA was extracted using RNeasy Mini Kit (QIAGEN) and analyzed using real-time qRT-PCR.

### Sphere formation assay.

Sphere formation assay was used to assess the in vitro self-renewal potential of stem cells. Cells in the logarithmic growth phase were digested and resuspended in serum-free medium supplemented with 20 ng/mL EGF, 10 ng/mL basic FGF, and 2% B27. The cells were then seeded on ultralow-attachment, 6-well plates (Corning). The spheres were cultured for 1 week, with the medium being replaced every 2 days. Spheres were pictured and observed using an inverted microscope.

### Xenograft cancer mouse model.

Adenovirus vectors carrying LncADAMTS9-AS2 (Adv-LncADAMTS9-AS2) and control adenovirus vectors were constructed at Genechem (Shanghai, China). In brief, HEK293T cells were cotransfected with an adenoviral shuttle vector carrying the ADAMTS9-AS2–encoding gene along with an auxiliary plasmid carrying most of the adenovirus genome.

The effect of *ADAMTS9-AS2* on tumor function was examined in vivo using nude female BALB/c mice aged 5 weeks (purchased from Beijing Weitonglihua Animal Center). A total of 2 × 10^6^ SK-N-Be2 cells were administered subcutaneously to the mice. One week after subcutaneous inoculation, mice were treated with virus. Adenovirus vectors carrying either the lncRNA ADAMTS9-AS2 gene or control were administered to the mice via injection in the tail vein. Four injections were administered every other day. All mice were sacrificed on the fourth weekend after adenovirus infection. All animal studies were conducted with approval of the Animal Ethical and Welfare Committee of Tianjin Medical University Cancer Institute and Hospital.

### Statistics.

Statistical analyses were conducted in SPSS 23.0 software. Pearson’s χ^2^ test was used to analyze clinicopathologic correlations. Differences between 2 groups or among 3 groups were compared using 1- or 2-way ANOVA and 2-tailed Student’s *t* test, respectively. All statistical tests were 2-sided, and *P* < 0.05 was considered statistically significant for all statistical tests.

### Study approval.

The study approval was obtained from the Ethical Committee on Scientific Research of Tianjin Medical University Cancer Institute and Hospital (E20210664). Participants gave written informed consent.

The mice used in this study were maintained in accordance with institutional animal care and use committee procedures and guidelines at Tianjin Medical University Cancer Institute and Hospital. All animal experiments were approved by the Animal Ethical and Welfare Committee of Tianjin Medical University Cancer Institute and Hospital.

### Data availability.

Values for all data points found in graphs are in the Supporting Data Values file. The sequencing data have been deposited in China National Center for Bioinformation under accession number HRA002064 (https://ngdc.cncb.ac.cn).

Further information can be found in Supplemental Methods.

## Author contributions

QZ and Yuanyuan Liu performed study conceptualization and designed the research. Yun Liu, JZ, FC, JL, YC, ZL, YG, and JY performed the research. Yun Liu and JZ generated the data. XD processed RNA-Seq data. Yun Liu, JZ, and FC prepared the figures. Yuanyuan Liu and Yun Liu wrote the manuscript. All authors read and approved the final manuscript.

## Supplementary Material

Supplemental data

Supporting data values

## Version 1 11/22/2023

Electronic publication
