# Supplementary material for: N6-methyladenosine–mediated overexpression of long noncoding RNA ADAMTS9-AS2 triggers neuroblastoma differentiation via regulating LIN28B/let-7/MYCN signaling
Source: JCI Insight. 2023 Nov 22;8(22):e165703. doi: 10.1172/jci.insight.165703 (PMC10721320; doi:10.1172/jci.insight.165703)
Supplement: Supplemental data [file jciinsight-8-165703-s125.pdf]

Supplemental Figures

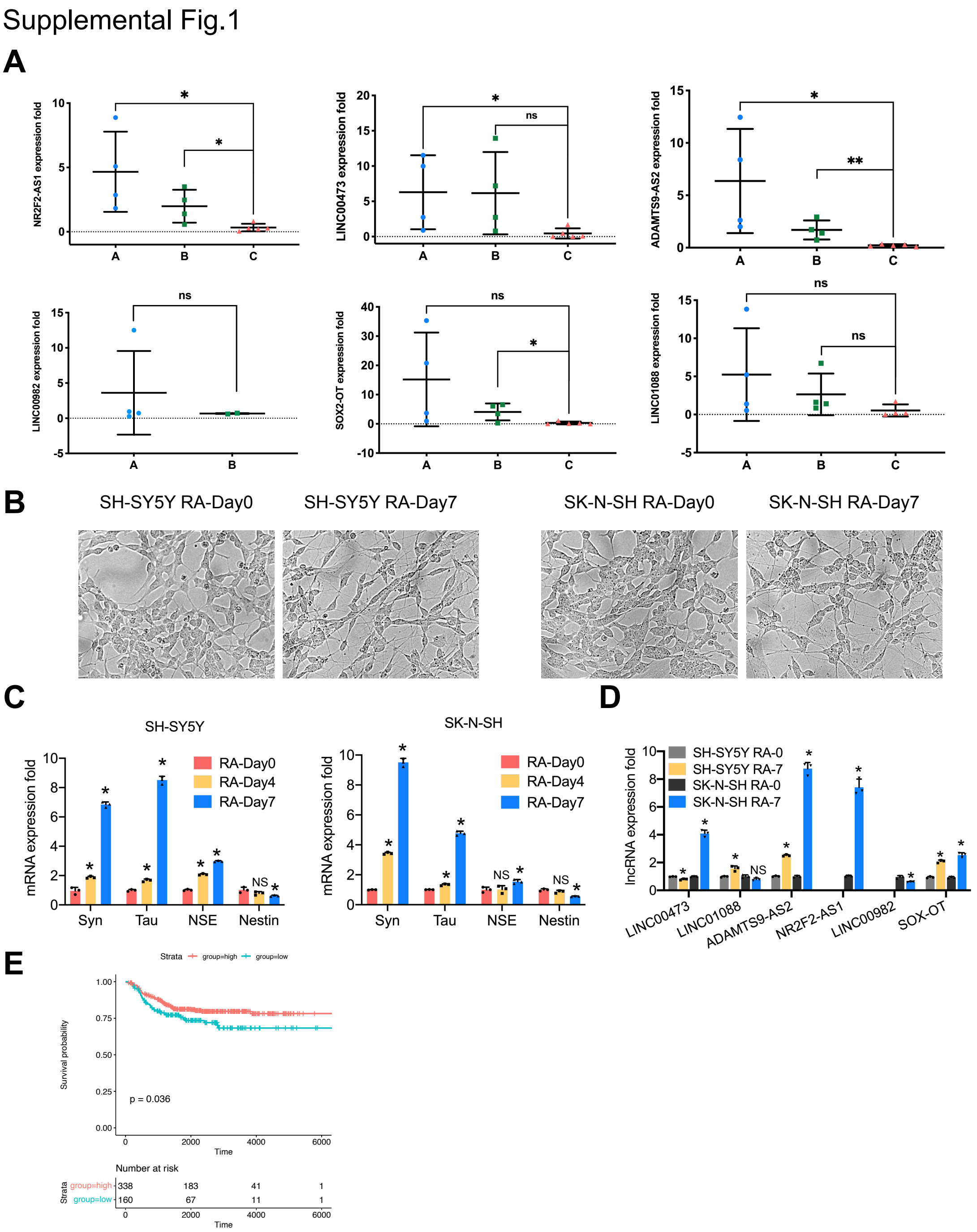

Supplemental Figure 1 *ADAMTS9-AS2* is differentially expressed in neuroblastoma.

A qRT-PCR analysis of relative expressions of candidate lncRNAs in neuroblastoma and intermixed ganglioneuroblastoma samples as compared with those in ganglioneuroma maturing subtype samples. High-risk and undifferentiated neuroblastoma (group C, n=5); low-risk and differentiating

ganglioneuroblastoma (group B, n=4); and ganglioneuroma maturing subtype (group A, n=4). Data are presented as mean ± SD. *P < 0.05; NS, not significant, two-sided Student's t test. **B** Morphological comparison between undifferentiated and differentiated SH-SY5Y cells between non-treatment and RA treatment group. **C** qRT-PCR was performed to validate the expression of *Synaptophysin* (*Syn*), *Tau*, *NSE* and *Nestin* expression level in SH-SY5Y and SK-N-SH with RA treatment. **D** qRT-PCR measurement of the candidate lncRNAs expression during RA induced neuronal differentiation of the SH-SY5Y and SK-N-SH cells. Data are presented as mean ± SD. *P < 0.05; NS, not significant, two-sided Student's t test. **E** Kaplan–Meier's correlation analyses between ADAMTS9-AS2 expression levels and overall survival in R2 database (Tumor Neuroblastoma - SEQC - 498 - RPM - eseqcnb1).

Supplemental Fig.2

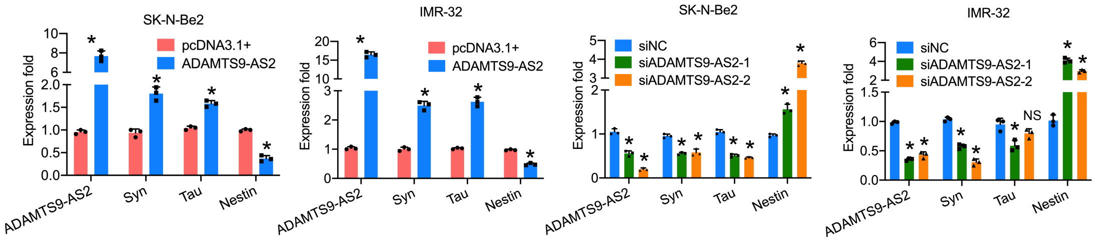

Supplemental Figure 2 *ADAMTS9-AS2* expression is critical for neuronal differentiation of neuroblastoma cells.

SK-N-Be2 and IMR-32 cells were transfected with control pcDNA3.1+, ADAMTS9-AS2 over-expression plasmid or control siRNA, ADAMTS9-AS2 siRNA-1, ADAMTS9-AS2 siRNA-2. *ADAMTS9-AS2*, *Syn*, *Tau* and *Nestin* RNA expression was examined by qRT-PCR, 18S rRNA was used as control. Data are presented as mean ± SD. *P < 0.05; NS, not significant, two-sided Student's t test or one-way ANOVA.

Supplemental Fig.3

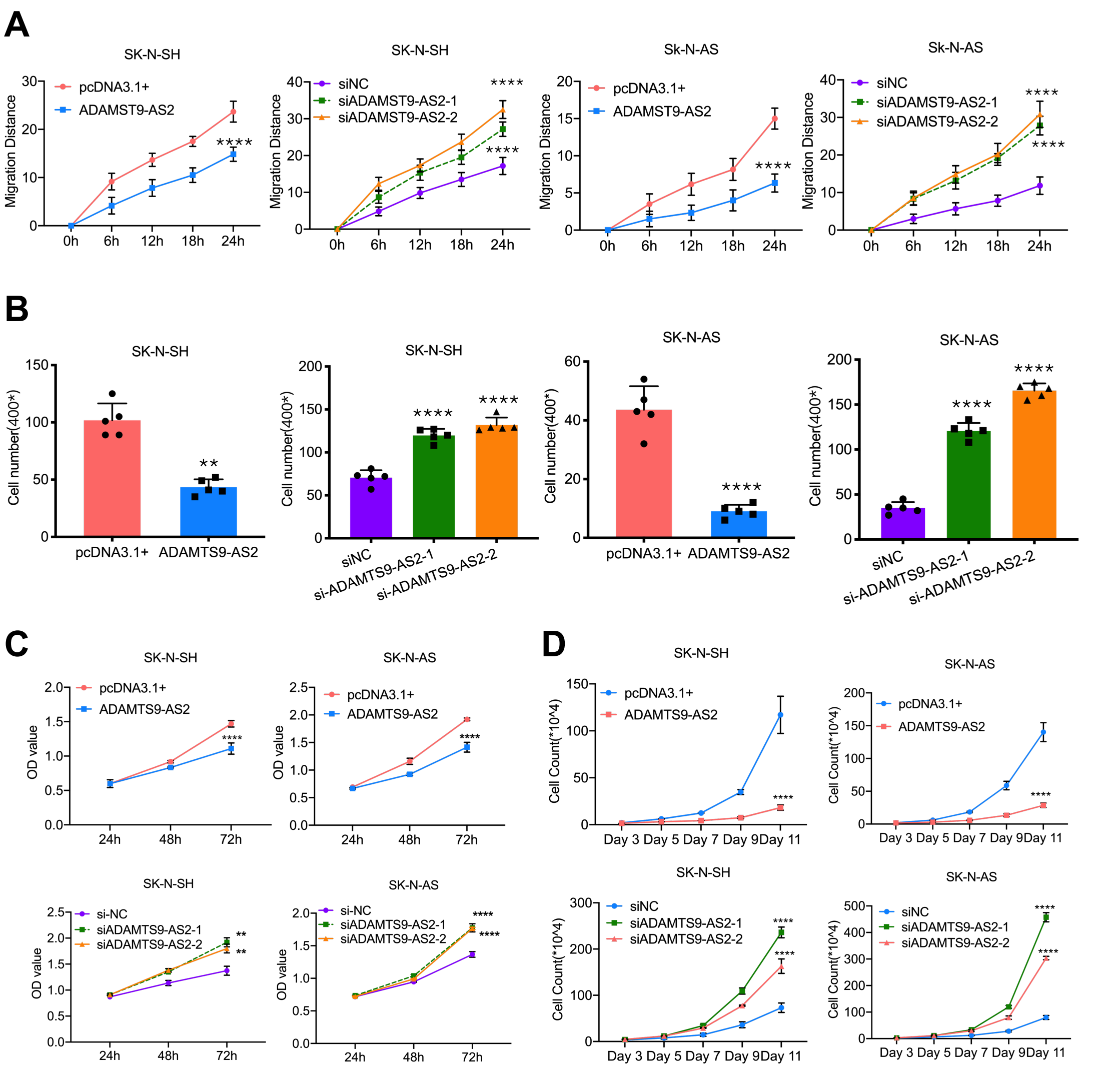

Supplemental Figure 3 LncRNA *ADAMTS9-AS2* harbors tumor suppressor properties.

A linear regression comparison of wound closure at regular intervals demonstrates the migration distance in up-regulation or down-regulation *ADAMTS9-AS2* of SK-N-SH or SK-N-AS cells. **B** The invasion assay of *ADAMTS9-AS2* over-expression and down-regulation cells was qualitatively recorded. **C** SK-N-SH and SK-N-AS ADAMTS9-AS2-overexpressing and knockdown cells were detected by CCK8 assay. **D** *ADAMTS9-AS2* up-regulation and down-regualtion SK-N-SH and SK-N-AS cells were detected by growth curve experiment. Data are presented as mean ± SD, n=3, * *P*<0.05. Data are presented as mean ± SD. **P* < 0.05; ** *P* < 0.01; **** *P* < 0.0001, one- or two-way ANOVA and Student's *t*-test.

Supplemental Fig.4

A

Protein	Peptide seq
LIN28B	GGGEEPGKLPEAEEESQVLR
	LFMEGFR
	EGEPVEFTFK
	VTGPGGSPCLGSER

Protein	Peptide seq
METTL17	HLPVEPEELQR
	FLENPDLSQTEEK
	LDGGFAAVSRAFHEIR
	QFLPVSPK

Protein	Peptide seq
ALKBH5	IDEVVSR
	FQFKPIRVSEPVLSLPVR
	SVLLPTHRR

B

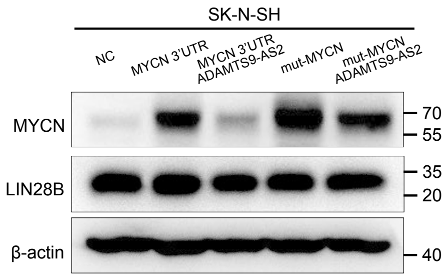

Supplemental Figure 4 LncRNA *ADAMTS9-AS2* interacts with LIN28B.

A Peptide sequences of LIN28B, METTL17, ALKBH5 interacting with ADAMTS9-AS2 in SK-N-Be2 cells identified in MS. **B** Examine expression of MYCN and LIN28B using MYCN ORF + intact 3'UTR, and MYCN ORF + let-7 site mutant 3'UTR in SK-N-SH cells.

Supplemental Fig.5

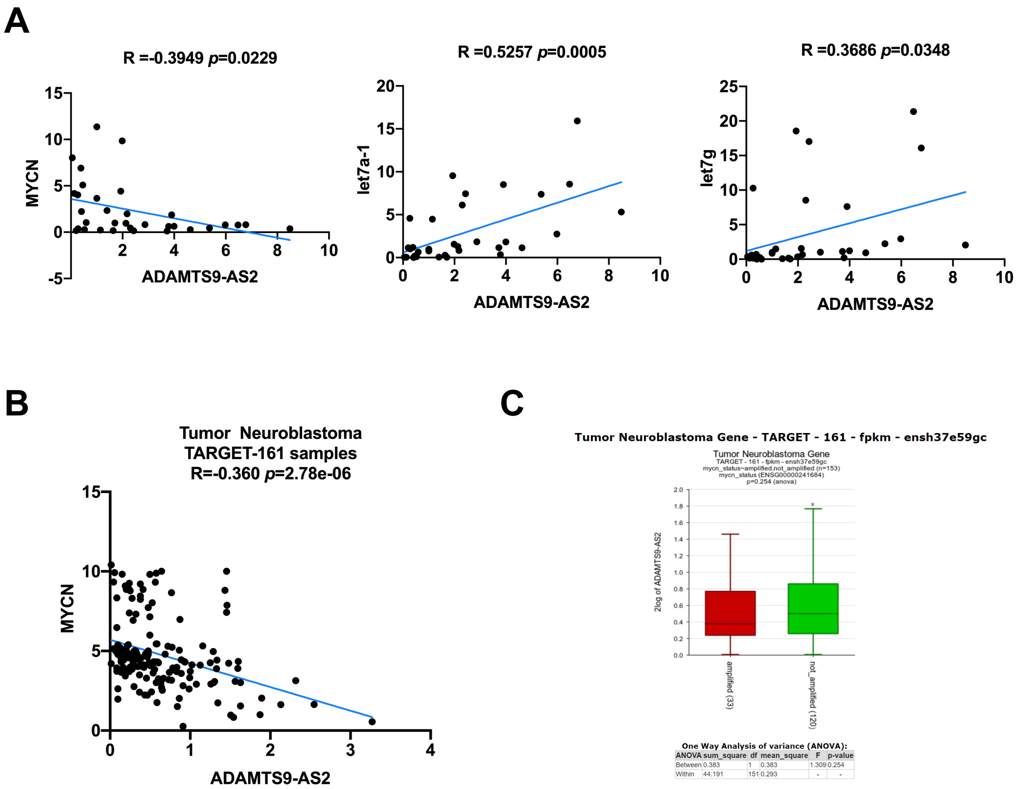

Supplemental Figure 5 LncRNA *ADAMTS9-AS2* interacts with LIN28B inhibit the association between LIN28B and *pri-let-7*.

A *ADAMTS9-AS2* and *let-7a-1*, *let-7g*, *MYCN* RNA expression were extracted from 33 human neuroblastoma tissue samples. Correlation between *ADAMTS9-AS2* and *MYCN*, *let-7a-1* and *let-7g* RNA expression was analyzed by two-sided Pearson's correlation. **B** Analysis the reverse correlation between *ADAMTS9-AS2* and MYCN in R2 database (Tumor Neuroblastoma Gene - TARGET - 161 - fpkm - ensh37e59gc). **C** Analysis the *ADAMTS9-AS2* expression between MYCN amplified and non-amplified samples in R2 database (Tumor Neuroblastoma Gene - TARGET - 161 - fpkm - ensh37e59gc).

Supplemental Fig.6

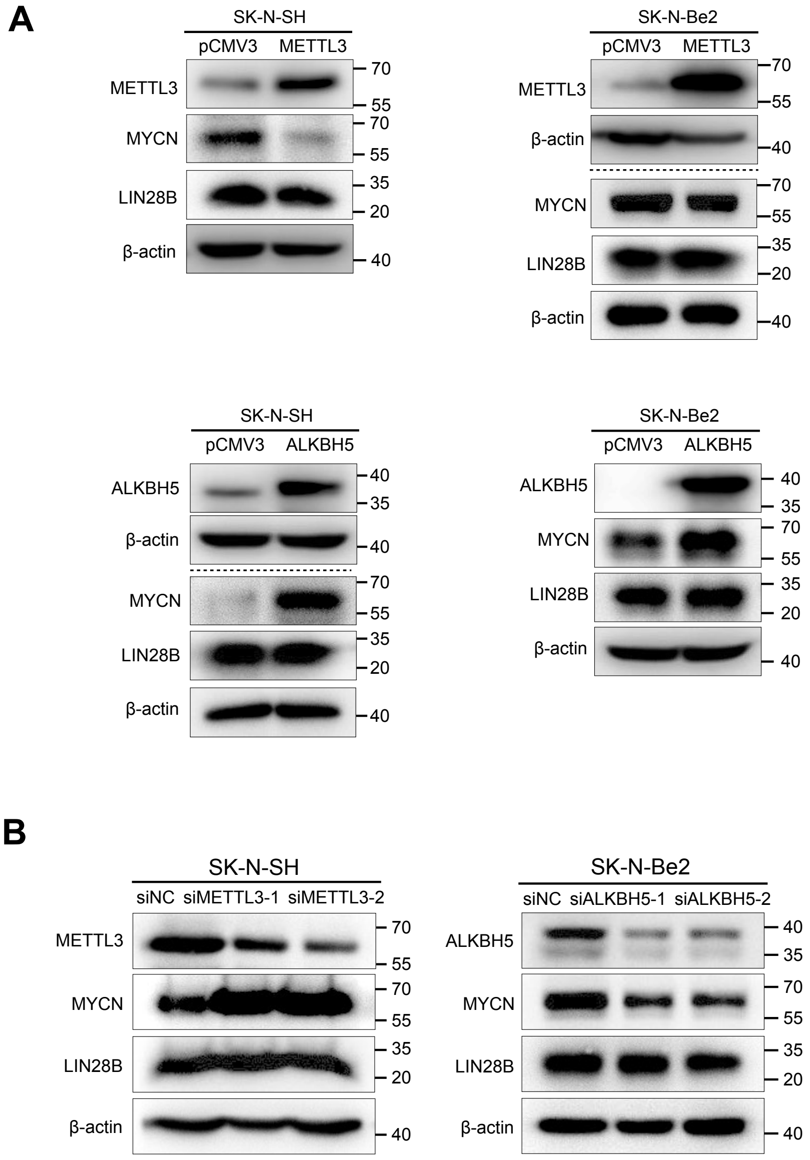

Supplemental Figure 6 m^{6}A modification is associated with *ADAMTS9-AS2* expression in NB cells.

A Western blot assay was performed to validate the expression level of MYCN and LIN28B in NB cells with transfected METTL3 or ALKBH5 overexpression plasmid. **B** The expression level of MYCN and LIN28B were assessed in SK-N-Be2 cells with ALKBH5 knockdown and SK-N-SH cells with METTL3 knockdown.

1. Supplemental Materials and Methods

1.1 RNA sequence

Total amounts and integrity of thirteen NB tumor samples' RNA were assessed using the RNA Nano 6000 Assay Kit of the Bioanalyzer 2100 system (Agilent Technologies, CA, USA). A total of 1 μg total RNA per sample was used as input material for the lncRNA library preparation. Strand-specific libraries were generated using NEBNext® UltraTM RNA Library Prep Kit for Illumina® (NEB, USA) following manufacturer's recommendations and index codes were added to attribute sequences to each sample. After the library is qualified, the different libraries are pooling according to the effective concentration and the target amount of data off the machine, then being sequenced by the Illumina NovaSeq 6000. Differential expression analysis between two sample groups was performed using the edgeR R package (3.24.3). The P values were adjusted using the Benjamini & Hochberg method. Significantly differentially expressed genes were defined as the following criteria: Corrected P<0.05 and absolute fold change > 2.

Total RNA was isolated from pcDNA3.1+ (n = 3) ADAMTS9-AS2 (n = 3) SK-N-SH cells. RNA samples were analyzed by RNA sequencing (Novogene, China) based on the manufacturer's protocols. Briefly, BGISEQ-500 platform was used to sequence the samples for subsequent generation of raw data. Genes significantly differentially expressed between pcDNA3.1+ control and ADAMST9-AS2 cells were selected based on fold change > 0 and P < 0.05 using the DEseq2 method. Functional pathway analysis was conducted using GO pathway enrichment analysis.

1.2 Western blot assay

RIPA lysis buffer (Thermo, USA) and bicinchoninic (BCA) protein assay kit (Thermo, USA) were used for protein extraction and concentration determination, respectively. Protein samples were separated by SDS-PAGE gel and transferred to PVDF membrane. The membranes were blocked with 5% skimmed milk in TBST at room temperature (RT) for one hour and buried in the primary antibody at 4℃ overnight. Then the membranes were incubated with the appropriate secondary antibodies for one

hour at RT. Immunoblots were exposed by ECL chemiluminescent detection system (Millipore, USA).

1.3 Immunofluorescence

For monolayer growing cells, the cells were plated into petri dishes with treated glass coverslips in advance and remove the cover glass after 24h. Washed three times with PBS, then the cells were fixed with 4% paraformaldehyde. After washing with PBS again, permeabilized with 0.25% Triton X-100 in PBS. Washed with PBS and blocked with 5% BSA. Then incubated overnight at 4°C with primary antibody. The second day, incubated with the appropriate secondary antibodies avoid light followed by washing with PBS. DAPI was used to counterstain cell nuclei. Dispensed one drop of Fluorescent Mounting Medium (Dako, Danish) onto slide and apply the glass coverslip over Fluorescent Mounting Medium to spread evenly over the specimen. Then use confocal laser scanning microscope to observe the image.

1.4 M6-methyladenosine mRNA immunoprecipitation (m6A-IP)

M6A-IP is a technology that uses M6A antibody to enrich methylated RNA and directly quantifies the enriched RNA using qPCR. The assay was performed by first isolating PolyA+ RNA from treated neuroblastoma cells. Protein A/G magnetic bead (Invitrogen, USA) and m6A antibody (Abcam, USA) are mixed in IP buffer (10 mM Tris-HCl pH 7.4, 150 mM NaCl, 0.1% NP-40) for 12h at 4 °C. PolyA+ RNA was incubated with the m6A-antibody-bound beads at 4 ℃ for 5 h. After immunoprecipitation, the bound RNA was isolated using RNeasy Mini Kit (QIAGEN, Germany) and quantitatively analyzed by carrying out real time PCR.

Supplemental tables

Table S1 Clinicopathological Information of Neuroblastoma Patients tissue samples for RNA-seq

	Sample A1	Sample A2	Sample A3	Sample A4	Sample B1	Sample B2	Sample B3	Sample B4	Sample C1	Sample C2	Sample C3	Sample C4	Sample C5
Age	≥18 months	<18 months	≥18 months	≥18 months	<18 months	≥18 months	≥18 months	≥18 months	≥18 months	<18 months	≥18 months	≥18 months	≥18 months
Gender	Female	Female	Male	Male	Female	Female	Female	Female	Male	Female	Female	Female	Female
Serum NSE Level (ng/mL)	≤ 400	≤ 400	≤ 400	≤ 400	≤ 400	≤ 400	≤ 400	≤ 400	>400	>400	≤ 400	>400	>400
Primary site	Abdomen	thorax	Abdomen	Abdomen	Abdomen	Abdomen	thorax	thorax	Abdomen	Abdomen	Abdomen	Abdomen	Abdomen
Maximal Tumor Size(cm)	> 5	> 5	> 5	< 5	< 5	> 5	> 5	> 5	< 5	< 5	> 5	> 5	> 5
Metastasis	Absent	Absent	Absent	Absent	Absent	Absent	Absent	Absent	Present	Present	Present	Present	Present
MYCN gene amplification	non-amplified	non-amplified	non-amplified	non-amplified	non-amplified	non-amplified	non-amplified	non-amplified	non-amplified	MYCN-amplified	MYCN-amplified	MYCN-amplified	MYCN-amplified
Diagnostic category	GN, maturing	GN, maturing	GN, maturing	GN, maturing	GNB intermixed	GNB intermixed	GNB intermixed	GNB intermixed	NB	NB	NB	NB	NB
Grade of differentiation	maturing subtype	maturing subtype	maturing subtype	maturing subtype	Differentiating	Differentiating	Differentiating	Differentiating	undifferentiated	undifferentiated	undifferentiated	undifferentiated	undifferentiated
Clinical stage of INSS	low-risk	low-risk	low-risk	low-risk	low-risk	low-risk	low-risk	low-risk	high-risk	high-risk	high-risk	high-risk	high-risk

Abbreviation: GN, ganglioneuroma; GNB, ganglioneuroblastoma; NB, neuroblastoma; INSS, International Neuroblastoma Staging System histology.

Table S2. Clinicopathological Information of Neuroblastoma Patients

	All	ADAMTS9-AS2 expression		*P value*
		Higher (n=61)	Lower (n=60)	
Gender				0.260
Male	64	30	34	
Female	57	31	26	
Age				0.397
≥18 months	89	46	43	
<18 months	32	15	17	
Primary site				0.044*
Abdomen, cervix	94	43	51	
Pelvis, thorax	27	18	9	
Maximal Tumor Size				0.149
>5cm	83	45	38	
≤5cm	38	16	22	
Metastasis				0.011*
Positive	63	25	38	
Negative	58	36	22	
Tumor Differentiation				0.016*
Low Grade	72	30	42	
High Grade	49	31	18	
Clinical stage of INSS				0.013*
1, 2	43	28	15	
3, 4	78	33	45	

Abbreviation: INSS International Neuroblastoma Staging System, * $P < 0.05$

Table S3. Sequences of the genes coding siRNAs for ADAMTS9-AS2 knockdown experiments.

Name	Target sequence
siADAMTS9-AS2-1	Sense (5'-3'): CAGAGACGCAGGUAUUUAUTT<br>Antisense (5'-3'): AUAAAUACCUGCGUCUCUGTT
siADAMTS9-AS2-2	Sense (5'-3'): CGGCUUUCAAGAUUGGAAUTT<br>Antisense (5'-3'): AUUCCAAUCUUGAAAGCCGTT

Table S4. Sequences of the DNA primers for qRT-PCR.

Name	Sequence (5'-3')
18S rRNA	Forward: GCTTAATTTGACTCAACACGGGA<br>Reverse: AGCTATCAATCTGTCAATCCTGTC
ADAMTS9-AS2	Forward: TTTACCATGCGCTGAGTGAG<br>Reverse: AAAGTTGCGTCATGCTTCGG
LINC00473	Forward: AAACGCGAACGTGAGCCCCG<br>Reverse: CGCCATGCTCTGGCGCAGTT
LINC01088	Forward: CGGCTTCCCCTTGAAGGAAT<br>Reverse: GGCCAGCTTGACTGTAGTGT
NR2F2-AS1	Forward: CTCTGGGAATCGTCCTGTATGC<br>Reverse: TGGTTTCCTGGTTCTCTGCC
LINC00982	Forward: TGCGAGCTTTGCTTTCTACTAA<br>Reverse: GGTCACTCTACAGAACTGGTCATTT
SOX2-OT	Forward: GTTCATGGCCTGGACTCTCC<br>Reverse: ATTGCTAGCCCTCACACCTC
Syn	Forward: ATTGTGCCAACAAGACCGAGAGT<br>Reverse: CAGGAAGATGTAGGTGGCCAGAG
Tau	Forward: GCGGCAGTGTGCAAATAGTCTACAA<br>Reverse: GGAAGGTCAGCTTGTGGGTTTCAA
Nestin	Forward: TGGCTCAGAGGAAGAGTCTGA<br>Reverse: TCCCCCATTTACATGCTGTGA
MMP9	Forward: TTCATCTTCCAAGGCCAATC<br>Reverse: CTTGTCGCTGTCAAAGTTCG
VCAM1	Forward: TATCTGCATCGGGCCTCACT<br>Reverse: AGGAAAAGAGCCTGTGGTGC
CCL2	Forward: AGCAAGTGTCCCAAAGAAGC<br>Reverse: CATGGAATCCTGAACCCACT
CCL5	Forward: TGCCCACATCAAGGAGTATTT<br>Reverse: TCTCTGGGTTGGCACACACTT

CCL7	Forward: ACCACCAGTAGCCACTGTCC<br>Reverse: GAGGAGCATCCCACAGTTTT
CD74	Forward: TGACCAGCGCGACCTTATCT<br>Reverse: GAGCAGGTGCATCACATGGT
BMPER	Forward: AGGACAGTGCTGCCCCAAATG<br>Reverse: TACTGACACGTCCCCTGAAAG
PTX3	Forward: CATCCAGTGAGACCAATGAG<br>Reverse: GTAGCCGCCAGTTCACCATT
LIN28B	Forward: TGATGCAGAAGATCACTCCGT<br>Reverse: ATATCCAAGGGGCTTCCCTCT
MYCN	Forward: CGCAAAAGCCACCTCTCATTA<br>Reverse: TCCAGCAGATGCCACATAAGG
SOX2	Forward: GGTTACCTCTTCCTCCCACTCC<br>Reverse: CCCTCCCATTTCCCTCGTTT
OCT4	Forward: GAAAGCGAACCAGTATCGAGAAC<br>Reverse: CCCCTGAGAAAGGAGACCCA
NANOG	Forward: ACCTATGCCTGTGATTTGTGG<br>Reverse: AGTGGGTTGTTTGCCTTTGG
GAPDH	Forward: TGCACCACCAACTGCTTAGC<br>Reverse: GGCATGGACTGTGGTCATGAG
U6 snRNA	Forward: CGCTTCGGCAGCACATATAC<br>Reverse: TTCACGAATTTGCGTGTCATC
pri-let-7a	Forward: AGGTGGTGGTAAGAGGGTGA<br>Reverse: TCCAGGGTGAATGGTGAAA
pri-let-7g	Forward: GCCAAGCCTCTGCTGTGA<br>Reverse: AGGGTGACGCCATCCTCT
let-7a-1	Forward: GCCGCTGAGGTAGTAGGTTGTA<br>Reverse: CAGAGCAGGGTCCGAGGTA
let-7g	Forward: GCCGCTGAGGTAGTAGTTTGTA<br>Reverse: TGGAGCCTGGGACGAGA

Table S5. Gene ontology (GO) analysis

Category	GOID	Description	GeneRatio	pvalue	padj	geneName
**BP**	GO:0019221	cytokine-mediated signaling pathway	12/58	2.052E-07	8.3527E-05	CCL2/CCL5/CCL7/CXCL8/HLA-B /IFI27/VCAM1/IFITM1/CD74/GBP2/HLA-F/LRRC15
**BP**	GO:0030335	positive regulation of cell migration	9/58	1.153E-05	0.0008922	CCL2/CCL5/CCL7/BDKRB1/MMP9/CEMIP/CXCL8/CD74/LRRC15
**BP**	GO:0070374	positive regulation of ERK1 and ERK2 cascade	5/58	0.0002389	0.00674368	CCL2/CCL5/CCL7/BMPER/CD74
**BP**	GO:0035821	modification of morphology or physiology of other organism	4/58	0.0005187	0.01172856	CCL5/KPNA7/SERPINB9/PTX3
**BP**	GO:0022409	positive regulation of cell-cell adhesion	5/58	0.0009437	0.01694515	CCL2/CCL5/GRAP2/VCAM1/CD74
